# Light‐Programmed Localized Supersaturation Enables Ambient Aqueous Synthesis of Highly Crystalline Metal–Organic Frameworks

**DOI:** 10.1002/adma.75080

**Published:** 2026-09-18

**Authors:** Yongdeok Ahn, Seunghyeon Jeong, Sun Ho Park, Juhyeong Cho, Nak Cheon Jeong, Daeha Seo

**Affiliations:** ^1^ Department of Chemistry POSTECH Pohang Republic of Korea; ^2^ Department of Physics and Chemistry DGIST Daegu Republic of Korea; ^3^ Department of Chemistry Education Gyeongsang National University Jinju Republic of Korea

**Keywords:** Cu_2_O photooxidation, HKUST‑1, interfacial crystallization, localized supersaturation, metal–organic frameworks

## Abstract

Crystalline materials, particularly metal–organic frameworks (MOFs), are essential for applications in catalysis, gas storage, and energy conversion, but conventional crystallization methods typically require high precursor concentrations or elevated temperatures, such as those provided by hydrothermal or solvothermal approaches. In this study, a self‐assembled nanoreactor was developed to achieve localized supersaturation via the photooxidation of Cu_2_O nanoparticles under visible light irradiation. Unlike conventional methods, this strategy enables HKUST‐1 crystallization at ambient temperature in water without external heating. By modulating the Cu^2+^ release response and ligand accessibility, we propose a two‐step process in which a metastable supersaturation regime forms a shell, followed by a labile supersaturation regime that favors predominantly inward crystal growth at or near the core‐shell interface. Transport perturbations, shell characterization, and a limiting reaction–diffusion analysis support this mechanistic interpretation, while local activities and the microscopic growth trajectory were not measured directly. X‐ray diffraction, Raman spectroscopy, and Brunauer–Emmett–Teller analyses confirmed the crystallinity and porosity of the resulting HKUST‐1. By generating localized supersaturation while maintaining dilute bulk conditions, this approach provides a programmable reaction environment for nucleation and growth, thereby opening a mild aqueous route to advanced functional materials.

## Graphical abstract

In the proposed model, a photoinduced nanoreactor uses Cu_2_O self‐photooxidation to generate localized supersaturation. A metastable supersaturation state first forms a shell, creating a spatially confined environment that restricts reactant exchange; continued Cu release then drives the local environment toward a labile regime that favors predominantly inward crystallization. This programmable approach uses spatial reactant localization rather than uniform bulk precursor loading or thermal activation to enable crystallization under mild aqueous conditions.

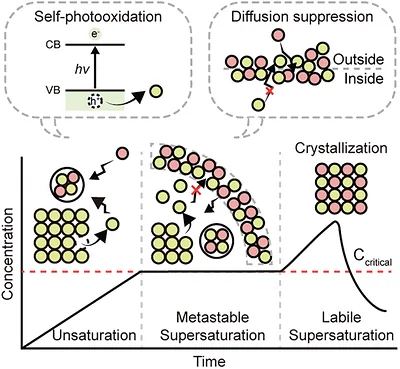

## 1 Introduction

Crystalline materials with well‐defined structures and physicochemical properties are crucial for applications in catalysis, energy conversion, and gas separation [1, 2, 3]. However, achieving highly crystalline and uniform materials often requires harsh synthetic conditions, such as high temperatures, solvothermal methods, and toxic solvents, which are energy‐inefficient and environmentally harmful [4, 5, 6]. A key strategy for synthesizing crystalline materials involves using supersaturation (*S*) to control nucleation and growth [7, 8, 9]. Based on classical nucleation theory and the LaMer model, upon reaching a critical concentration (*C*_critical_), burst nucleation is triggered, followed by precursor depletion, which shifts the system to a growth‐dominated regime [10, 11]. Although methods based on the generation of *S* in the bulk facilitate nucleation, they often require high secondary building unit (SBU) concentrations, elevated temperatures, or additional reagents to increase the reaction constant (*k*), rendering them inefficient and unsustainable (Figure 1a). To overcome these limitations, recent approaches have focused on the generation of localized *S* to confine nucleation within restricted regions. Template‐mediated and encapsulation systems have been investigated; however, their scalability is limited as they cannot selectively control the degree of *S* [12, 13, 14, 15, 16].

**FIGURE 1:**
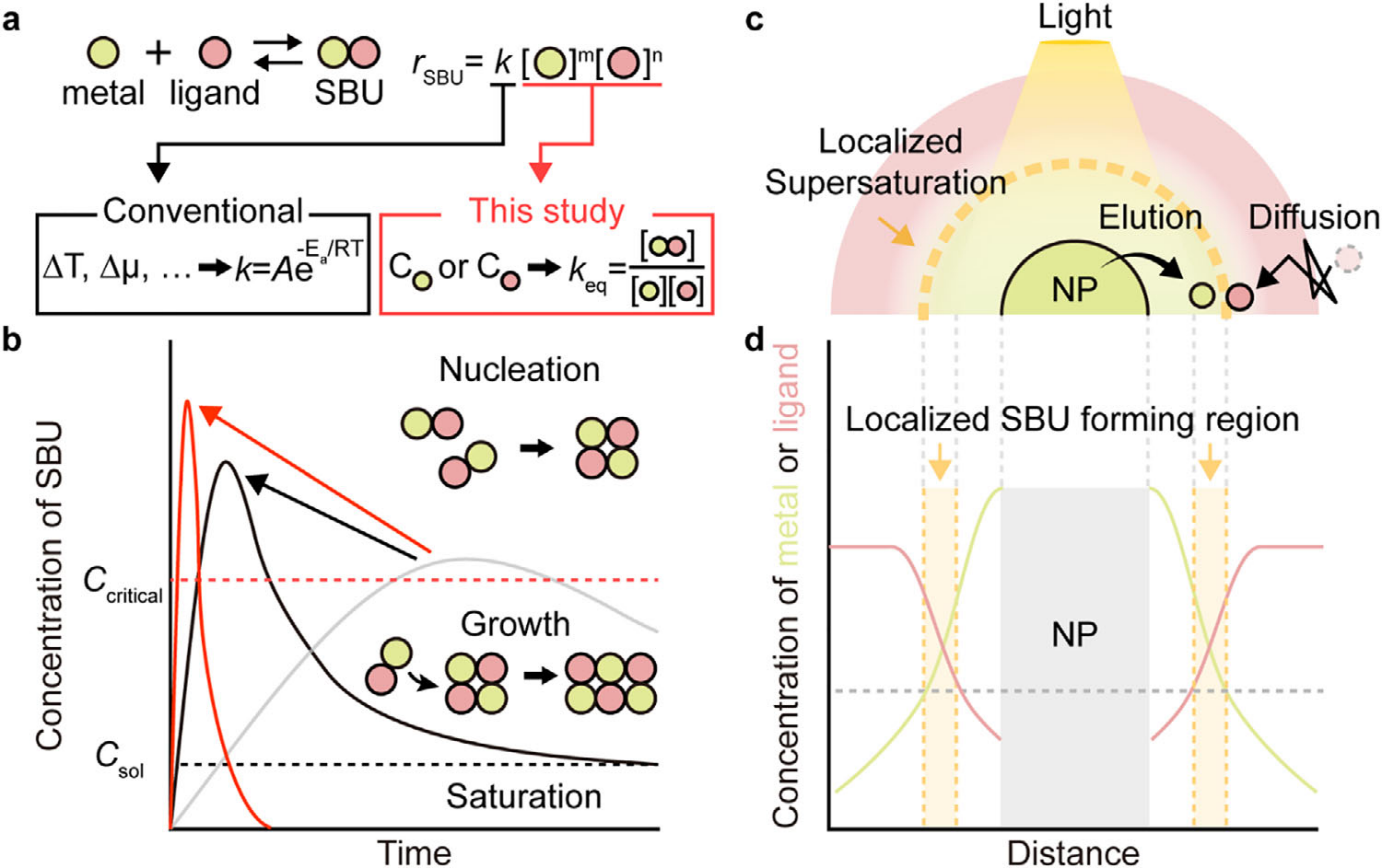
Schematic representation of the localized supersaturation strategy for crystal growth driven by Cu_2_O NP‐mediated self‐photooxidation. (a) Reaction scheme for SBU formation and comparison between conventional strategies (black) and the approach adopted in this study (red) that relies on controlling the precursor concentration via self‐photooxidation. (b) Time‐dependent SBU concentration profiles based on LaMer‐type behavior. The red curve (this study) shows rapid *S* and nucleation, followed by fast SBU depletion, which limits further growth. The black curve (conventional methods) shows extended growth, while the gray curve (spontaneous nucleation under ambient conditions) shows sluggish kinetics with limited nucleation. (c) Generation of Cu^2+^ by the irradiated Cu_2_O NP creates a local Cu‐rich region near the NP. While Cu^2+^ diffuses outward, external ligands diffuse inward, producing spatial overlap of the precursors. (d) Proposed spatial concentration profiles of Cu^2+^ and ligands showing a localized SBU‐formation zone within the model. The curves and profiles are conceptual and do not represent measured local activities.

In this study, we developed a self‐assembled nanoreactor driven by the self‐photooxidation of Cu_2_O nanoparticles (NPs) in water under visible‐light irradiation [17, 18]. In contrast to conventional thermal synthesis (>50°C), the approach allows SBU and crystal formation to occur at ambient temperature and atmospheric pressure through self‐photooxidation‐induced localized delivery of Cu species at the particle interface (Figure 1b) [19]. Unlike bulk synthesis methods, the proposed strategy based on the self‐photooxidation of Cu_2_O facilitates the local control of Cu^2+^ release and induces crystal growth under mild conditions without the use of external catalysts (Figure 1c). The rapid generation of Cu‐containing species creates a Cu‐rich environment around the NPs and favors SBU formation. The spatial overlap of metal precursors and organic ligands favors SBU formation within restricted regions (Figure 1d). Our proposed mechanistic model comprises three stages (Figure S1): i) the fast elution of metal ions and modulation of the ligand approach to form SBUs; ii) formation of a heterogeneous shell within a metastable *S* region (*S*_meta_, [Cu^2+^]_eq_ < [Cu^2+^]_local_ ≤ *C*_critical_) at the Cu_2_O NP surface; and iii) interface‐driven growth within a labile *S* region (*S*_labile_, *C*_critical_ < [Cu^2+^]_local_) at the core–shell interface as transport becomes restricted, favoring well‐crystallized materials [20, 21]. Leveraging the intrinsic photoactivity of Cu_2_O enables the precise control of Cu^2+^ release in the confined environment, facilitating efficient nucleation and high‐quality crystal formation. This approach departs from conventional paradigms by enabling the nanoscale spatial programming of a chemical reaction, with confined reaction domains proposed to influence the final crystal form. Here, *S*_meta_ and *S*_labile_ are operational descriptors of the observed sequence; local activities and the microscopic growth pathway were not measured directly.

## 2 Results and Discussion

### 2.1 Theoretical Basis for Nucleation via Local Supersaturation

To evaluate the proposed approach, we employed HKUST‐1, a Cu‐based metal–organic framework (MOF) with high porosity and a well‐defined structure, as a model system [22]. Its nucleation and growth were investigated to assess whether localized *S* can enable crystalline framework formation under mild aqueous conditions.

Crystallization processes are strongly dependent on how and when nuclei are formed, as illustrated in the LaMer plot. First, the precursor concentration gradually increases owing to chemical reactions or the continuous addition of soluble species. Beyond *C*_critical_, nucleation occurs in a short burst, with numerous nuclei forming simultaneously [23, 24, 25]. These nuclei rapidly deplete the SBUs, reducing their concentration to below the nucleation threshold. At this stage, further nucleation halts, and crystal growth dominates. At higher *S*, nucleation proceeds more rapidly, resulting in a greater number of smaller and more uniform particles. However, maintaining a high *S* throughout a solution volume is challenging, especially when the precursor is expensive or prone to side reactions. Thus, a localized approach that concentrates the precursor at specific sites can drive nucleation while limiting bulk reagent loading.

From a thermodynamic perspective, the crystallizing phase must reach a negative volume free energy (Δ*G_v_*) before nucleation (Figure S2a) [26]. This transition occurs when critical supersaturation *S*_critical_ is exceeded, at which point the solid phase becomes thermodynamically favorable. Spontaneous nucleation is unlikely below *S*_critical_ but becomes feasible above it. Upon the formation of a nucleus, its stability is governed by classical nucleation theory (Figure S2b). The free energy (Δ*G*) of a nascent nucleus peaks at a critical radius (*r*_c_) [27]. Clusters smaller than *r*_c_ tend to dissolve, whereas those exceeding *r*_c_ can reduce their Δ*G* by continuing to grow. Because *r*_c_ decreases with increasing *S*, even small clusters can overcome the activation barrier (*E*_a_), functioning as stable nuclei with sufficiently high *S* [28, 29, 30].

To reduce the aforementioned barrier, an alternative approach to the conventional strategy of increasing the overall temperature involves generating localized high‐*S* regions (Figure S2c). The relationship between *S* and *r*_c_ is expressed as *r*_c_ = 2γv_m_/(*k*_B_ *T*ln*S*), where *γ* is the surface energy, v_m_ is the molecular volume of the crystallizing phase, and *k*_B_ is Boltzmann's constant. Thus, increasing *S* above *S*_critical_ reduces *r*_c_ without requiring a large thermal input. High local *S* can lower the nucleation barrier in a manner analogous to increasing temperature. In this study, instead of heating the entire system, we leveraged a self‐assembled nanoreactor that concentrates the precursor near a specific interface, thereby creating a localized high‐*S* zone.

### 2.2 Design Strategy for Localized Supersaturation via the Self‐Photooxidation of Cu_2_O Nanoparticles

Building upon the theoretical understanding of *S* dynamics, we aimed to develop a controlled experimental system in which Cu^2+^ release could be precisely modulated to achieve localized *S*. This led to a systematic investigation of the effects of a localized *S*_meta_ region on nucleation and crystal growth. Instead of uniformly increasing the SBU concentration across the entire solution volume, we exploited the formation of a self‐assembled shell triggered by Cu^2+^ release in response to the self‐photooxidation of Cu_2_O NPs under visible‐light irradiation to create a localized *S*_meta_ region around the NP (Figure 2a). Within the proposed model, shell formation under mild conditions requires sufficient local precursor enrichment (*S*_meta_) near the NP surface [31, 32].

**FIGURE 2:**
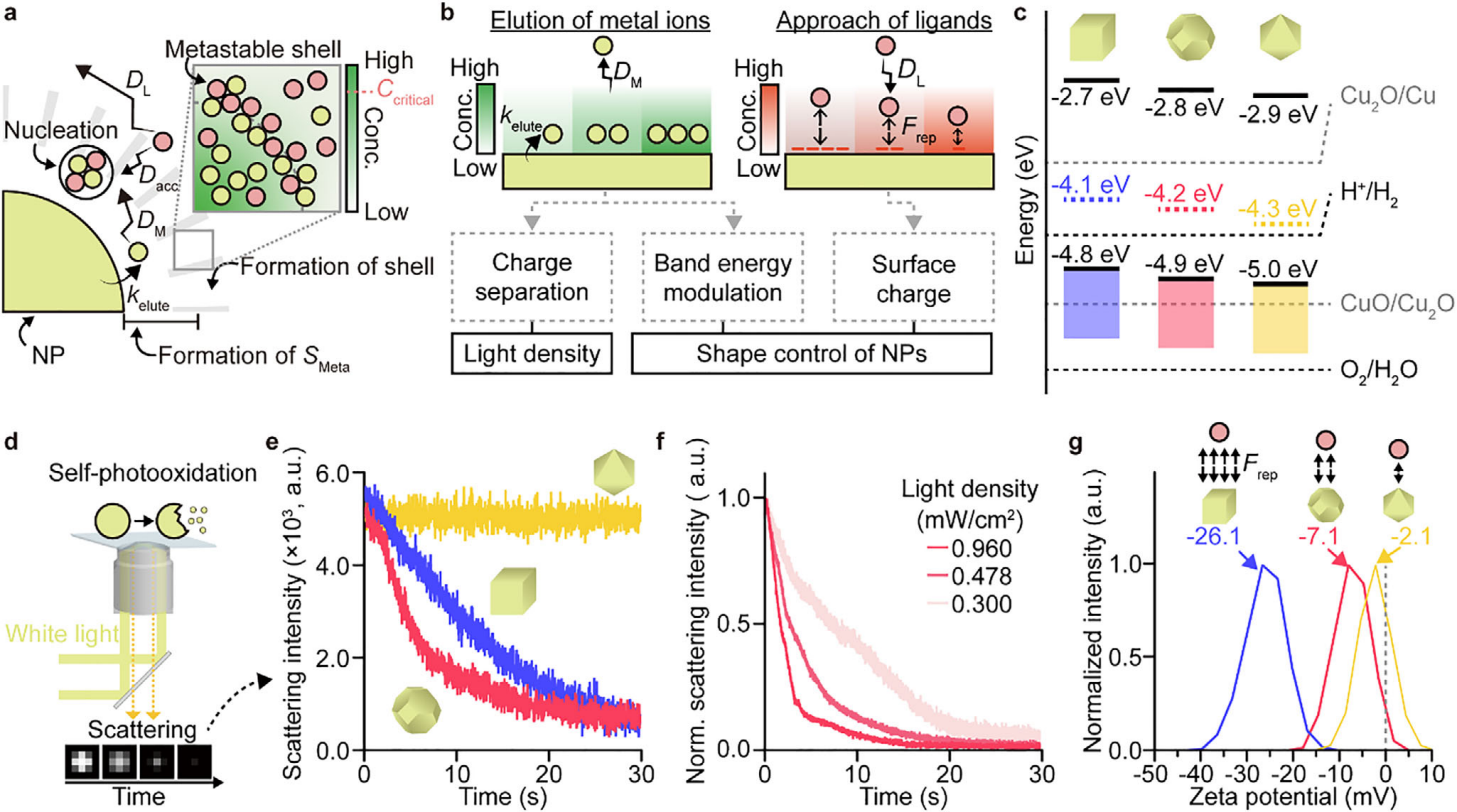
Control of *k*_elute_ and *D*_acc_ via the morphological control of NPs to form shells. (a) Schematic of the proposed formation of an *S*_meta_ region at the NP surface that drives partial shell formation. The shell confines a localized supersaturated volume, while the external solution remains in an unsaturated state (*S*_un_). (b) Schematic of the optimization of *k*_elute_ and *D*_acc_ by adjusting the NP surface environment. (c) Band‐energy level for each NP morphology. (d) Self‐photooxidation setup using visible light to evaluate the relative *k*_elute_ based on the NP morphology. (e) Time‐resolved scattering intensity for NPs with different morphologies. (f) Time‐resolved scattering intensity of t‐Cu_2_O under different incident irradiances. (g) Zeta potential results for the various NPs, illustrating the proposed contribution of electrostatic repulsion to ligand accessibility and shell formation.

In the proposed model, formation of a confined *S*_meta_ region relies on two key factors (Figure 2b). First, a sufficiently high Cu^2+^ release rate (*k*_elute_) is required to maintain local Cu enrichment against diffusion loss (*D*_M_) from the particle surface [33]. Second, sufficient ligand accessibility (*D*_acc_) is required: 1,3,5‐benzenetricarboxylic acid trivalent anion (BTC^3−^), the ligand for HKUST‐1, must approach the NP surface to initiate shell growth despite electrostatic repulsion (*F*_rep_) from the negatively charged Cu_2_O surface. By controlling *k*_elute_ and *F*_rep_, the local metal‐ and linker‐species can cross a kinetic threshold at which SBU formation rate (*k*_formation_) exceeds the crystal dissolution rate (*k*_dissolution_). We use *S*_meta_ to denote this shell‐forming condition; the local activities and elementary rate constants were not measured directly.

### 2.3 Cu^2+^ Release Rate Control via Self‐Photooxidation

Cu^2+^ release is primarily driven by the accumulation of photogenerated holes, which can be regulated through either light intensity modulation or NP morphological control. Furthermore, the local concentration of the ligands surrounding the NPs can influence *D*_acc_ by altering the NP surface charge. *D*_acc_ is governed by the interplay between ligand diffusion (*D*_L_) and *F*_rep_, which can be finely tuned by controlling the NP morphology.

To systematically investigate the effects of *k*_elute_ and *F*_rep_, the following experiments were performed using shape‐controlled Cu_2_O NPs. The NP morphology determines the formation of crystallographic facets, each of which exhibits different stability and terminal atomic species. These differences influence the band energy levels of the surface lattice and considerably affect the surface charge characteristics. We focused on tuning *k*_elute_ and *F*_rep_ by adjusting the NP morphology and varying the intensity of the incident light required to trigger the self‐photooxidation of the NPs. First, we prepared Cu_2_O NPs with three different shapes, cubic (c‐Cu_2_O), truncated octahedral (t‐Cu_2_O), and octahedral (o‐Cu_2_O), by varying the polyvinylpyrrolidone (PVP) concentration (Figure S3). The three types of Cu_2_O NPs feature different combinations of (100) and (111) crystal facets and exhibit distinct energy levels, as characterized using ultraviolet photoelectron spectroscopy and Tauc plot analysis (Figures 2c and S4). Among them, c‐Cu_2_O possesses the highest valence‐band energy level, indicating its superior hole accumulation capability. However, t‐Cu_2_O, with (100) and (111) facets, exhibits accelerated hole accumulation via internal charge transfer across the facet junctions (Figure S5) [34].

Real‐time observation using optical microscopy confirmed the unique structural features of each type of NP (Figure 2d,e) [35]. As expected, t‐Cu_2_O exhibited the fastest self‐photooxidation. Notably, no significant difference in scattering intensity between light‐on and light‐off conditions was observed for o‐Cu_2_O. SEM analyses after visible‐light irradiation revealed self‐photooxidation trends similar to those observed via optical microscopy (Figure S6a). Quantitative analyses based on inductively coupled plasma–mass spectrometry (ICP‐MS) performed under irradiation with various light intensities confirmed that self‐photooxidation can be precisely tuned via photonic control (Figure S6b). These findings indicate that *k*_elute_ can be modulated by controlling NP morphology. Furthermore, we observed self‐photooxidation in real time at the single‐particle level by varying the intensity of the light incident on t‐Cu_2_O, which exhibited the highest *k*_elute_ (Figure 2f). The rate of self‐photooxidation increased considerably with light intensity. These results show that the NP morphology and incident light intensity control the measured Cu‐release response, a key input to localized SBU formation.

### 2.4 Ligand Accessibility Control via Surface‐Charge Modulation

Next, we investigated the role of *F*_rep_ in controlling ligand accessibility. Owing to its low water solubility (2.6 g L^−1^ at 25°C), H_3_BTC was completely deprotonated to BTC^3−^ by triethylamine (TEA), resulting in more homogeneous dispersion and increased reactivity [36, 37]. Further, the strong negative charge of BTC^3−^ results in electrostatic repulsion with the negatively charged surfaces of the Cu_2_O NPs. Zeta potential measurements confirmed substantial differences between the surface charges of c‐Cu_2_O (−26.1 mV), t‐Cu_2_O (−7.05 mV), and o‐Cu_2_O (−2.09 mV) NPs (Figure 2g). Under thermodynamically stable surface conditions, the (100) facet exhibits the highest density of terminal oxygen atoms, leading to the most negatively charged surface. Based on the Coulombic model, *F*_rep_ between a negatively charged NP and a ligand with a charge of −3 was approximated by determining the effective NP charge *Q*_NP_ using *Q*_NP_ = 4*πε*_0_ *ε*_r_ *R*ζ, where ζ is the zeta potential, *R* is the radius of the NP, *ε*_r_ is the relative permittivity of the solvent, and *ε*_0_ is the permittivity of free space [38]. The corresponding Coulombic interaction energy at a separation *r* scales as *F*_rep_ ∝ ζ*q*_ligand_/*r*, providing a relative measure of electrostatic repulsion when all other conditions are identical [39]. By normalizing the nominal repulsion at the c‐Cu_2_O surface to unity, the illustrative values for the t‐Cu_2_O and o‐Cu_2_O surfaces were estimated to be ∼0.27 and 0.08, respectively. Here, ζ is used only as a nominal electrokinetic input because it is measured at the slipping plane rather than at the true surface. Derjaguin–Landau–Verwey–Overbeek theory (DLVO) and the Poisson–Boltzmann descriptions therefore support an electrostatic contribution to *D*_acc_, while residual PVP, hydration, and the evolving surface chemistry during photooxidation may also influence ligand accessibility [40, 41, 42]. Consequently, surface electrostatics are considered an important factor influencing *D*_acc_ on the NP surfaces. This electrostatic description is necessarily simplified because residual PVP may affect colloidal dispersity and surface hydration, while the surface evolves during self‐photooxidation through facet‐dependent hole accumulation and Cu^2+^ release. Thus, electrostatic repulsion is considered a major factor governing ligand accessibility rather than its sole determinant. Consistent with this interpretation, XPS results show the presence of residual PVP on t‑Cu_2_O and o‐Cu_2_O (indicated by N/Cu ratios approximately 3.5 and 3.7 times that of c‐Cu_2_O, despite the threefold difference in PVP loading during synthesis). However, two independent comparisons showed that this residual PVP does not govern shell formation. PVP‐free c‐Cu_2_O formed no shell, despite the absence of significant PVP steric hindrance, and the two PVP‐bearing samples with nearly identical residual PVP concentrations formed a robust and thin partial shell, respectively (Figure S7). The impacts of PVP loading and ζ on shell‐formation outcomes for each morphology are summarized in Table S1.

### 2.5 Formation of a Core–Shell Structure in the *S*_meta_ Regime

Next, we investigated the roles of *k*_elute_ and *D*_acc_ in promoting shell formation on Cu_2_O NPs as an essential step toward creating a self‐assembled nanoreactor. To control *k*_elute_ precisely by adjusting the light intensity, the photo‐assisted reaction was conducted using a laboratory‐built reaction vessel (Figure S8). Transmission electron microscopy (TEM) analyses revealed remarkable differences among the three NP types under identical conditions (Figure 3a). For c‐Cu_2_O, no shells formed around the NPs; instead, the materials grew at a distance from the NP surface. Based on the proposed electrostatic interpretation, the highly negative NP surface charge restricts access of BTC^3−^ (Table S2), hindering shell formation. In contrast, thin shells with thickness of tens of nanometers grew on the o‐Cu_2_O surfaces. However, further shell growth was not observed due to the low *k*_elute_. Nonetheless, the smaller negative charge (−2.09 mV) of o‐Cu_2_O facilitated sufficient ligand diffusion for a certain degree of shell formation. Finally, t‐Cu_2_O exhibited robust heterogeneous shell growth, as confirmed by TEM images. In addition, small crystals were observed near the core–shell NPs, indicating that certain precursors led to successful crystallization, but others generated only metacrystallized products [43]. With this model, this mixed outcome corresponds to the *S*_meta_ regime rather than a fully crystalizing state. Even under the same *D*_acc_ condition, decreasing *k*_elute_ alone by reducing the intensity of the incident light was insufficient for shell formation. Under these conditions, an unidentified bar‐like material was observed. Together, these results indicate that sufficiently high *k*_elute_ and *D*_acc_ generate a local *S*_meta_ environment and enable crystal formation under mild reaction conditions. Furthermore, we performed time‐dependent thermal measurements to confirm that the heating effect of visible‐light irradiation over the entire reaction process was negligible (Figure S9).

**FIGURE 3:**
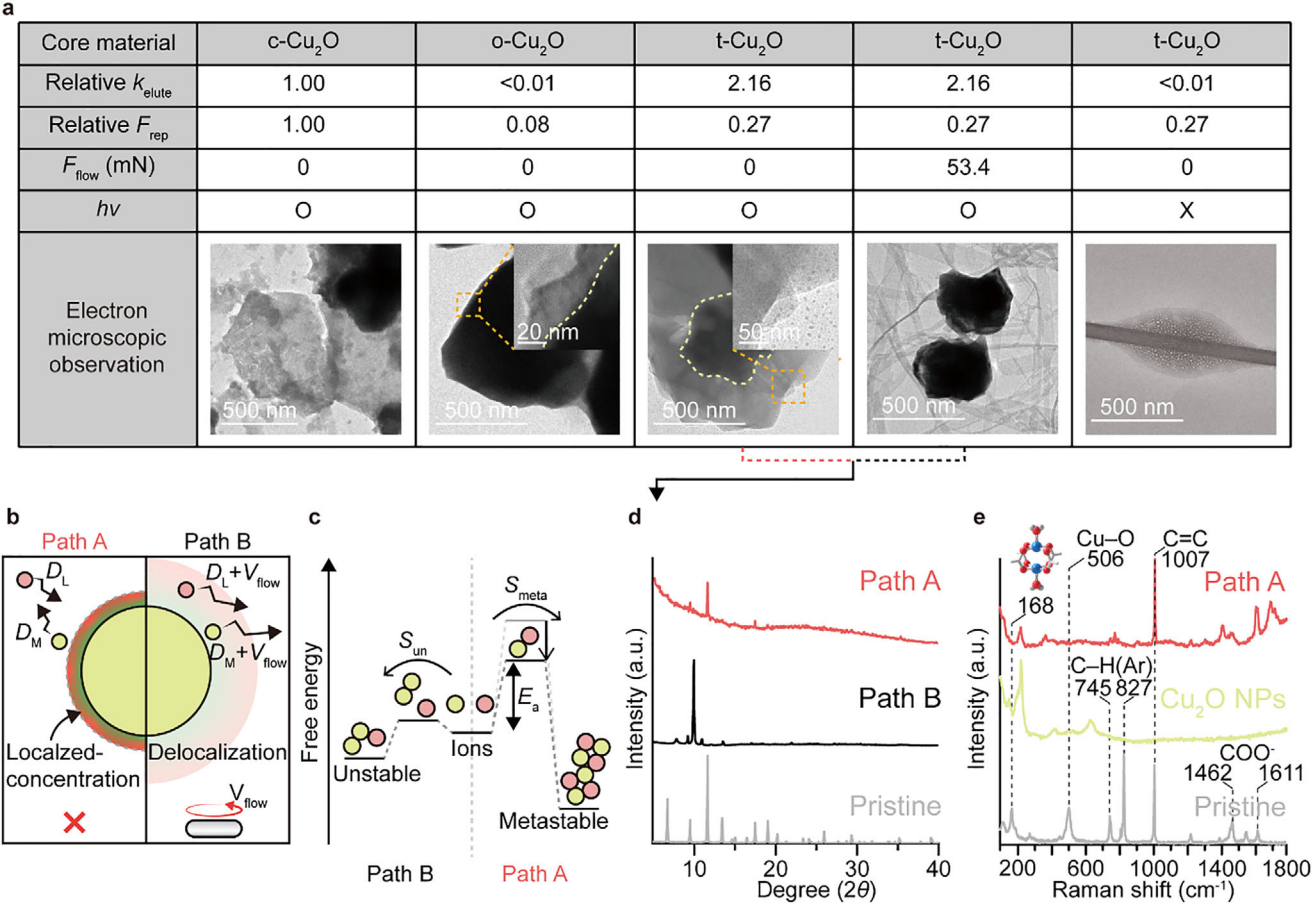
Proposed shell formation in the *S*_meta_ region generated by the self‐photooxidation of Cu_2_O NPs. (a) Comparison of NP types in terms of relative *k*_elute_, *F*_rep_, *F*_flow_, and *hv* (top table), and TEM images showing shell formation (bottom). (b) Schematic comparing the reaction conditions that yield a localized concentration gradient (no stirring, Path A) versus a more diffuse concentration gradient (stirring, Path B). (c) Schematic of the two reaction pathways: Path A (*S*_meta_ regime) and Path B (*S*_un_ < *S*_meta_ regime). (d) XRD patterns of the products obtained via Path A (core–shell), Path B, and pristine HKUST‐1. (e) Raman spectra of the Path A product, t‐Cu_2_O, and pristine HKUST‐1.

To examine the importance of maintaining a local concentration gradient, the reaction was performed under vigorous stirring as a transport‐perturbation condition (Figure 3b) [44, 45, 46]. For example, stirring at 700 rpm produced strong convective mixing, with an estimated flow force (*F*_flow_) of 53.4 mN and a flow velocity (*V*_flow_) of 0.37 ms^−1^, thereby enhancing mass transport and reducing the persistence of particle‐associated concentration gradients (Supporting Note S1). This condition was therefore used to test whether disruption of the particle‐associated concentration gradient alters shell formation and the subsequent crystallization pathway.

Under quiescent conditions (Path A, Figure 3c), Cu species generated by self‐photooxidation remain localized near the NP surface, where their spatial overlap with approaching BTC^3^^−^ favors the formation of shell‐bearing intermediates. Vigorous stirring (Path B, Figure 3b) introduces convective transport (*V*_flow_) in addition to molecular diffusion, dispersing both metal and linker species and thereby reducing their local accumulation near the particle surface (Figure 3c). Based on our model, this transport perturbation shifts the system away from the shell‐forming *S*_meta_ regime toward a more delocalized, unsaturated condition (*S*_un_), in which the nucleation barrier remains comparatively high and the metastable shell‐bearing intermediate is not favored (Path B) [47, 48]. Thus, Paths A and B provide experimentally accessible conditions that differ primarily in the persistence of the particle‐associated concentration gradient. Without stirring, the shell‐bearing product showed weak HKUST‐1 reflections by x‐ray diffraction (XRD) (Figure 3d).

To validate the formation of the core–shell structure, we performed Raman and Fourier transform infrared (FTIR) spectroscopic analyses on the core–shell NPs (Figures 3e and S10). Raman bands were observed at 220 and 615 cm^−1^ for core–shell NPs obtained via Path A, which were assigned to the second‐order Raman‐allowed and T_1u_ modes of Cu_2_O, respectively [49, 50]. These features indicate a change in the local structure of Cu_2_O during shell formation. In particular, the band at 329 cm^−1^ lies close to the A_2u_ mode reported in the 308–350 cm^−1^ region of Cu_2_O [51]. This is a silent mode forbidden in ideal cuprite that becomes Raman‐active only when the lattice symmetry is locally broken. Therefore, the presence of this peak indicates defect‐activated modes generated as the Cu_2_O surface is disrupted during shell formation. In contrast, the FTIR spectrum of the core–shell NPs contain the characteristic carboxylate bands of pristine HKUST‐1, indicating that BTC^3^^−^ is coordinated to copper through the same carboxylate binding mode as in the HKUST‐1 framework. While the Raman analysis shows that the underlying Cu_2_O core is structurally disrupted, the FTIR results show that BTC–Cu carboxylate coordination is already established at the shell. Notably, the core–shell NPs differ from pristine HKUST‐1 (obtained via conventional synthesis) in their overall spectral characteristics, indicating that they are transient particles during the transformation from t‐Cu_2_O NPs to HKUST‐1 crystals. Thus, the spectroscopic data identify the shell‐bearing particle as an intermediate in which Cu–BTC coordination has developed, but the HKUST‐1 framework is not yet fully established.

To clarify the physical and chemical properties of the intermediate shell, we complemented the Raman and FTIR analysis with x‐ray photoelectron spectroscopy (XPS) and high‐resolution TEM with fast‐Fourier‐transform analysis (HR‐TEM/FFT). The XPS results (Figure S11) indicate that the shell surface contained predominantly Cu^+^ with only a minor Cu^2+^ component, as quantified from the Cu2p_3/2_ shake‐up satellite‐to‐main‐peak area ratio (0.087, compared with ∼0.5 for reference CuO). In addition, a distinct carboxylate (COO^−^) contribution in the C 1s spectrum confirms partial BTC^3^^−^ coordination at a Cu^+^‐rich surface (Supporting Note S2, Tables S3 andS4). The HR‐TEM/FFT images, obtained selectively at the shell region of individual particles (Figure S12), show that the shell is predominantly amorphous, with only minor, locally dispersed crystalline domains; the FFT pattern of one such domain matched that of crystalline Cu(OH)_2_. Combined with the weak HKUST‐1 reflections in the XRD pattern (Figure 3d) and the absence of the 168 cm^−1^ Cu–Cu paddlewheel mode in the Raman spectrum, these results indicate that the shell is not a single phase but a heterogeneous, largely amorphous composite comprising residual Cu^+^ oxide matrix, crystalline domains (e.g., Cu(OH)_2_), and non‐paddlewheel Cu–carboxylate species. Within our crystallization model, we assign this shell‐bearing intermediate to the *S*_meta_ regime, in which formation of the HKUST‐1 framework remains kinetically limited and lower‐barrier species can develop concurrently [52].

The Raman spectra of the Path B product further distinguish between stirred and quiescent pathways (Figure S13). Spectra acquired from spatially distinct regions of the sample differ, indicating that the loss of confinement opened several concurrent coordination pathways rather than directing the reaction to a single product. Across these regions, three distinct local environments were resolved. In some regions, the Cu_2_O bands at 219 cm^−1^ are no longer observed, and a new band appears near 284 cm^−1^, indicating a structural change in the NPs; in others, the Cu_2_O bands are largely retained with the same 284 cm^−1^ feature, consistent with locally defective but largely intact Cu_2_O. In a third type of region, bands at 731, 816, 1008, 1447, and 1589 cm^−1^ reveal BTC^3^^−^ coordination to Cu, with peak positions distinct from those of pristine HKUST‐1 (COO– at 1467 and 1553 cm^−1^). These positions are instead comparable to those reported for the Cu–BTC coordination polymer Cu(BTC)·3H_2_O [53], in which the selection between phases is known to depend on the local Cu^2+^/BTC^3^^−^ ratio and the degree of ligand deprotonation. The divergence of this product from HKUST‐1 indicates that localization in the quiescent system biases the reaction toward the HKUST‐1 pathway.

Beyond its crystallographic identity, the Path B product is also chemically distinct from the quiescent core–shell intermediate at the surface level. Under identical XPS acquisition and processing conditions and after removing any dependence on absolute intensity by comparing intra‐spectrum area ratios, the carboxylate‐to‐aliphatic carbon ratio (O–C═O at ∼288 eV / C–C at ∼284.8 eV) of the core–shell intermediate is approximately twice that of the stirred product (COO–/C–C ≈ 0.10 vs ≈0.05), whereas the O 1s envelopes of the two samples are nearly indistinguishable (Figure S11). This indicates that the two pathways differ not in their oxygen environment but in the extent of trimesate coordination to copper: the quiescent, confinement‐controlled pathway advances ligand coordination roughly twofold further than the stirred pathway. This quantitative surface‐chemical distinction shows that stirring opens an alternative reaction pathway and that localization more effectively advances the metal‐linker coordination required for framework assembly.

### 2.6 Nanoreactor‐Mediated *S*_labile_ and Crystallization

Although the formation of the initial shell occurred owing to the existence of the *S*_meta_ region, continued self‐photooxidation altered the local chemical environment, which, in the proposed model, drives the system toward the *S*_labile_ regime with rapid nucleation and directed crystal growth. The heterogeneous shells on the NP surfaces were inherently unstable, resulting in continuous dissociation at the solvent–shell and core–shell interfaces. In our proposed model, this instability is attributed to the tens‐of‐nanometer‐thick transition layer of the MOFs, which favors recrystallization at or near the core–shell interface [54, 55, 56]. Within this confined space, the *S* dynamics are driven by two key processes: sustained Cu^2+^ supply via self‐photooxidation, which is generated by hole accumulation in the core domain, and a restricted outward transport through the shell, which increases the particle‐associated Cu inventory (Figure 4a).

**FIGURE 4:**
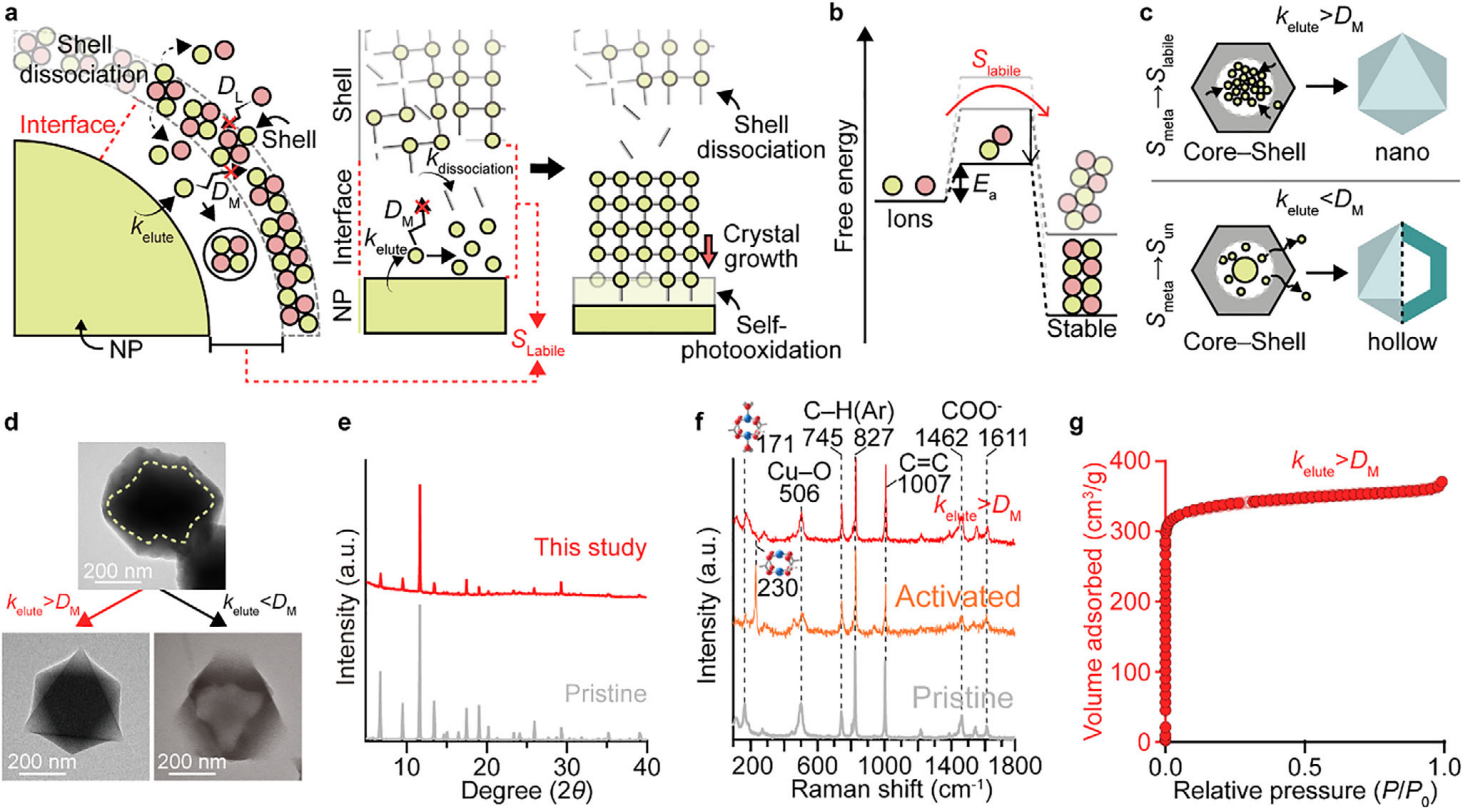
Proposed crystallization pathway in the *S*_labile_ regime within the self‐assembled nanoreactor. (a) Schematic of the self‐assembled nanoreactor at the core–shell interface. (b) Schematic of the proposed reaction pathway in the *S*_labile_ regime, in which the *E*_a_ for crystallization is lowered, favoring crystal reconfiguration. (c) Schematic of the effect of Cu‐release (*k*_elute_)‐dominant and diffusive‐loss‐(*D*_M_) dominant conditions on product morphology. (d) TEM images of core–shell (top), nano‐HKUST‐1 (left), and hollow‐HKUST‐1 (right) particles formed under different light conditions (that modulate *k*_elute_) after the formation of the core–shell structure. The overlaid yellow dashed line indicates the core–shell interface. (e) XRD patterns of nano‐HKUST‐1 formed in this study and pristine HKUST‐1. (f) Raman spectra of nano‐HKUST‐1, activated nano‐HKUST‐1, and pristine HKUST‐1. (g) N_2_ adsorption isotherm of nano‑HKUST‑1.

The proposed transport‐restricting role of the shell is supported by three observations that probe exchange between the particle‐associated region and the bulk. First, the dissolved Cu detected in the bulk during shell formation remained below the level expected for free dispersal of the released copper (Figure S14), indicating retention or consumption of Cu near the particle. Second, external metal cations introduced after shell formation were not detected in the final crystals by EDS elemental mapping, which resolved Cu but not the added ions under the tested conditions (Figure S15). However, this experiment did not include a positive incorporation control, quantitative detection limit, or bulk compositional analysis; thus, the absence of a detectable signal cannot establish that these cations were completely excluded from the crystals. Third, when structurally distinct linkers BDC^2−^ (2D) or DHTP^4−^ (1D) were introduced after shell formation but before the completion of crystallization, the product remained the 3D octahedral morphology of HKUST‐1, comparable to that formed without linker addition (Figure S16). Because BDC^2−^ and DHTP^4−^ direct the assembly to connectivity and dimensionality distinct from HKUST‐1, substantial participation of these externally added linkers would be expected to perturb the octahedral habit or produce a different phase. Their addition did not alter the particle morphology noticeably, supporting their limited participation in framework assembly, although low‐level incorporation cannot be excluded without compositional analysis of the digested product.

Taken together, the Cu‐release, external‐cation, and linker‐addition experiments support a transport‐restricting role of the shell that limits exchange between the particle‐associated region and the bulk. Within our model, this confinement favors retention of released Cu and sustains the localized supersaturation required for the transition from *S*_meta_ to *S*_labile_, thereby promoting predominantly inward crystallization from the shell. The present data do not support this as the exclusive microscopic pathway, and local dissolution–reprecipitation may also contribute to the observed growth.

Consequently, in the proposed model, the localized region evolves from *S*_meta_ to *S*_labile_ as continued Cu release and restricted transport increase the local driving force and lower the nucleation barrier. This facilitates rapid nucleation and the formation of highly crystalline products that quickly stabilize into a well‐ordered phase (Figure 4b). In addition, BTC^3−^ transport within the nanoreactor region is comparatively slow through shell dissociation or gradual permeation across the pores or defects of the shells. Considering the reaction orders with respect to Cu^2+^ (+1.10) and BTC^3−^ (−1.14), the resulting Cu‐rich, linker‐limited profile favors the growth of HKUST‐1 [57].

Within this framework, the shell plays two roles in the reaction process: it promotes the development of an *S*_labile_ environment that favors nucleation and shifts the kinetic balance toward *k*_formation_ > *k*_dissociation_, thereby producing well‐crystallized HKUST‐1 (Figure 4c). Considering the reaction order with respect to the precursors, the localized enrichment of Cu species within the shell domain, together with limited BTC^3−^ availability, further facilitates HKUST‐1 growth. With continued light irradiation, nano‐octahedral crystals (nano‐HKUST‐1) formed rapidly, as confirmed via TEM and SEM analyses (Figures 4d and S17). These octahedral crystals were collected using an ethanol‐based extraction process (Figure S18 and Supporting Note S3, Table S5). Control experiments confirmed that this step recovers, rather than generates, the crystalline product. Applying the same ethanol‐based extraction process without light and quenching with acetic acid did not yield HKUST‐1, whereas purely physical isolation by ultrafiltration recovered pre‐formed HKUST‐1 crystals, albeit in low yield and with partial dissociation, owing to the absence of protonation that stabilizes the framework (Figures S19 and S20). Notably, an octahedral product was observed within 1 min in ex situ ensemble samples only for shell‐bearing NPs but not for those without a shell. This temporal correspondence supports a central role for the shell, although it is not a continuous single‐particle observation. The size distribution of the octahedral crystals closely matches that of the t‐Cu_2_O crystals, indicating that their final dimension was determined by the initial size of the Cu_2_O NPs (Figure S21). Together, the temporal and size correspondence supports particle‐templated, predominantly inward growth, although local dissolution–reprecipitation cannot be excluded.

HKUST‐1 is produced via the reaction of 3Cu^2+^ + 2BTC^3−^ → Cu_3_(BTC)_2_. The relevant thermodynamic *S* therefore depends on the activities of both the Cu‐ and BTC^3^^−^‐containing species rather than on [Cu^2+^] alone. Because neither a reliable *K*_sp_ nor the corresponding activity coefficients are available under the present aqueous‐TEA condition, we use the local‐to‐bulk Cu inventory ratio as a proxy for relative precursor enrichment and retain S_meta_ and S_labile_ as operational regime labels. *S*_meta_ denotes the metastable shell‐forming regime in which the local enrichment is sufficient for intermediate formation, but rapid nucleation remains kinetically suppressed. *S*_labile_ denotes the labile regime in which the local driving force becomes sufficient for nucleation. We define the apparent boundary between these regimes as *C*_critical_. Previous studies of HKUST‐1 crystallization place the relevant nucleation and growth concentrations broadly in the sub‐mM to few‐mM range [58, 59]. We therefore use this range as an order‐of‐magnitude reference for *C*_critical_ in the present system. Notably, these concentrations do not represent a measured critical threshold. A complete relative‐enrichment and upper‐bound analysis is provided in Supporting Note S2.

Within this framework, predominantly inward, confined growth is quantitatively consistent with the limiting local‐inventory calculation. Using the measured Cu^2+^ release flux (Figure 2f) and particle geometry, the pre‐shell open‐boundary model gives ∼9.5 µM at the NP surface. After shell formation, an idealized closed‐boundary case with negligible leakage and consumption gives a geometric upper bound of a few tens of molar; the aqueous solubility of simple Cu(II) salts provides a practical external ceiling of ∼5 M, which lies well above the sub‐mM to few‐mM *C_c_*_ritical_ range discussed above. Based on this calculation, the local Cu inventory can therefore reach up to ∼50–100‐fold that of conventional synthesis conditions, with an estimated accumulation time of ∼10 s. The spatiotemporal evolution of this limiting concentration field is visualized in Movie S1. However, the Cu(OH)_2_‐like domains and non‐paddlewheel Cu–carboxylate species observed within the shell (Figures S11 and S12) indicate that competing consumption may lower the free Cu activity. Thus, these numerical values represent model‐derived upper bounds rather than concentrations, free‐Cu^2^^+^ activities, or thermodynamic supersaturations experimentally measured from the Cu_2_O/H_3_BTC/TEA system. Considering these limits, the calculation provides quantitative support for confinement‐enhanced local Cu inventory and the proposed transition from *S*_meta_ to *S*_labile_ (Supporting Note S2).

In the self‐assembled nanoreactor, *k*_elute_ modulation also plays a critical role in determining the resulting crystal structure. Upon reducing the light intensity, *k*_elute_ remained lower than *D*_M_, suppressing further SBU nucleation and crystal growth within the shell and leading to the formation of hollow crystals (H‐HKUST‐1) via shell dissociation. To validate this hypothesis, the light intensity was gradually varied in the range of 0.960–0.3 mW cm^−2^ during the reaction, resulting in hollow structures as expected (Figures 4d and S22). Although H‐HKUST‐1 was not uniformly produced on a large scale, its emergence supports the proposed link between the light‐intensity profile and crystal morphology.

To fabricate more‐uniform hollow structures, we modulated the reaction kinetics by applying pulsed‐light irradiation (5 s on/off cycles) and by varying the reaction temperature to tune the reaction constants governing Cu^2+^ release, diffusion, and crystallization, resulting in aggregated and plate‐like products, respectively (Figure S23). The pulsed condition repeatedly interrupted and reversed the shell dissociation process that produces the hollow interior, whereas the uniform kinetic suppression did not selectively preserve the *k*_elute_ < *D*_M_ condition required for hollowing. Together, these results indicate that the formation of uniform hollow structures requires a synchronized, spatially uniform intensity ramp‐down delivered to the entire NP ensemble.

Further TEM analysis provided additional insights. First, ex situ TEM images of independently prepared samples collected at successive irradiation times indicate that conversion was substantially complete within 1 min in the sampled ensemble, which is very close to the complete self‐photooxidation time of a single NP (∼1 min) (Figure S24). Because these images sample different particles at predetermined time points, they establish the ensemble time scale rather than the growth trajectory of an individual particle. The similar ensemble and photooxidation timescales support the proposed sequence. Second, crystal dissociation occurred when the products were left in the reaction solution without quenching, indicating that the transient layer underwent continuous degradation. This observation shows that crystal dissolution contributes to the supply of BTC^3−^ into the nanoreactor and further crystal growth. Third, reducing the light intensity inhibited internal growth within the shell, as predicted by the model. However, the variation in the reaction initiation times across individual NPs and the rapid progression of crystallization within a few tens of seconds made it difficult to precisely determine the time at which the light intensity should be reduced. Consequently, large‐scale production of H‐HKUST‐1 is limited by the difficulty in achieving precise temporal control owing to the short reaction time (<1 min).

XRD and FTIR analyses were performed to confirm the formation of nano‐HKUST‐1 in the self‐assembled nanoreactor. The XRD pattern of the as‐synthesized HKUST‐1 shows diffraction peaks at 2*θ* = 6.7°, 9.6°, 11.7°, and 13.4°, which are characteristic of pristine HKUST‐1 obtained via conventional synthesis, confirming the structural integrity of the product (Figure 4e). Furthermore, the FTIR spectrum exhibits asymmetric carboxylate stretching bands at 1600–1500 cm^−1^, benzene ring vibration bands at 1400–1300 cm^−1^, and a Cu–O stretching band at 700–750 cm^−1^, which are in excellent agreement with the reported FTIR results for HKUST‐1, confirming the formation of this material (Figure S25). The Raman spectrum of nano‐HKUST‐1 shows carboxylate stretching bands at 1611 and 1641 cm^−1^, an aromatic C = C stretching band at 1007 cm^−1^, aromatic C–H stretching bands at 745 and 827 cm^−1^, and a Cu−O stretching band at 506 cm^−1^, which are characteristic of pristine‐HKUST‐1 (Figure 4f) [60]. Furthermore, a Cu−Cu vibrational band is observed at 171 cm^−1^, indicating the coordination of water molecules to the Cu centers. To induce water dissociation, the Raman laser power was increased from 1.0 to 3.0 mW, resulting in a blue shift of the Cu−Cu vibrational band to that of the open state at 230 cm^−1^ [61]. This shift confirms that HKUST‐1 was successfully synthesized with accessible open metal sites.

To evaluate the pore volume and integrity of the framework, we conducted Brunauer–Emmett–Teller N_2_ adsorption measurements (Figure 4g). The HKUST‐1 synthesized in the *S*_labile_ environment exhibited N_2_ adsorption values of 350–400 cm^3^g^−1^, similar to those of nanoscale MOFs reported in the literature [62]. Further, a Type‐I isotherm is observed, indicating the formation of a well‐defined, predominantly microporous MOF framework. These results demonstrate that HKUST‐1 synthesized in the *S*_labile_ environment retains the essential framework characteristics of pristine HKUST‐1, including its crystallinity and porosity. Together with the diffraction and spectroscopic data, these measurements confirm the successful synthesis of crystalline, microporous HKUST‐1 under ambient aqueous conditions.

Unlike conventional methods to prepare HKUST‐1, we obtained identical crystalline phases using water as the solvent at ambient temperature and atmospheric pressure. Although our study did not directly determine the absolute thermodynamic supersaturation and its quantitative difference from hydrothermal synthesis, our localized supersaturation model suggests that confinement increases the particle‐associated inventory and thereby provides the driving force required for nucleation under mild conditions.

### 2.7 Generality and Limitations of the Localized Supersaturation Strategy

To examine whether this strategy extends beyond HKUST‐1, we applied the t‐Cu_2_O photooxidation approach to two additional Cu‐MOF linkers under fully deprotonated conditions: 1,4‐benzenedicarboxylate (BDC^2−^, associated with Cu‐MOF‐2) and 2,5‐dihydroxyterephthalate (DHTP^4−^, associated with Cu‐MOF‐74). Like BTC^3^^−^, BDC^2−^ formed a core–shell intermediate followed by crystalline domains in selected regions and particles (Figures S26a and S27), consistent with the proposed shell‐mediated crystallization sequence. For BDC^2−^, the core–shell intermediate exhibited Raman bands attributable to both the Cu_2_O core (145, 219, 405, and 626 cm^−1^) and coordinated BDC^2−^ (864, 1137, 1433, and 1609 cm^‑1^), whereas the extracted product showed bands at 866, 1142, 1449, and 1614 cm^−1^, which agreed with reported spectra of crystalline Cu–BDC materials [63, 64]. Together with HR‐TEM/FFT, these measurements identify local Cu–BDC‐like crystalline domains and provide proof‐of‐concept evidence that the proposed sequence can extend beyond BTC^3^^−^. However, the bulk phase identity, including phase fraction, purity, yield, and porosity, of the recovered product was not measured mainly because the ethanol‐based extraction procedure was optimized for HKUST‐1 and recovered less than 2% of the corresponding mass for the BDC‐derived product, leaving insufficient material for bulk characterization.

In contrast, DHTP did not form a detectable core–shell intermediate on t‐Cu_2_O, as determined by HR‐TEM (Figures S26b and S28) [65]. Although the Raman spectra of the DHTP samples showed Cu–carboxylate coordination, the characteristic band assigned to crystalline Cu‐MOF‐74 (the C–O stretching mode near 1273 cm^−1^) was absent, and no shell was detected by HR‐TEM. Thus, Cu–DHTP coordination occurred without detectable formation of the shell‐bearing intermediate. Based on our proposed interpretation, the lower near‐surface accessibility of DHTP inhibits shell nucleation, allowing released Cu species to coordinate with DHTP outside the shell‐forming interfacial region. This behavior parallels that observed for c‐Cu_2_O (Figure 2); however, limited linker accessibility prevents shell formation. The present measurements do not resolve the exact location or microscopic pathway of Cu–DHTP coordination. Collectively, the BDC^2−^ and DHTP^4−^ results provide proof‐of‐concept evidence that the proposed shell‐mediated crystallization strategy can extend beyond H_3_BTC while identifying linker accessibility as a practical constraint on its scope.

Within the model, the relevant requirement is electrostatic accessibility: a fully deprotonated anionic linker must approach the negatively charged Cu_2_O surface to nucleate the confining shell (Supporting Note S4). In a mean‐field description, the near‐surface concentration is described by the Boltzmann factor

$$C_{suf}/C_{bulk}=\exp\left(-W\right),W=\left|z\right|e\left|\psi_{0}\right|/\left(k_{B}T\right)$$

We use the measured zeta potential (ζ = −7.05 mV) as a nominal effective‐potential input for this illustrative calculation. Because ζ is measured at the slipping plane, it is not equated with the true surface potential ψ_0_. This approximation gives surface‐to‐bulk concentration ratios of 0.58, 0.44, and 0.33 for H_2_BDC, H_3_BTC, and H_4_DHTP, respectively. The ratios are model‐derived ratios rather than measured near‐surface concentrations, but their ordering—BDC^2^^−^ > BTC^3^^−^ > DHTP^4^^−^—illustrates the expected monotonic effect of linker charge near a negatively charged interface.

The reference *W* = 1, corresponding to *C*_surf_/*C*_bulk_ ≈ 1/*e* (≈ 0.37), denotes the condition at which the nominal electrostatic penalty equals *k_B_T*. It is used here as a characteristic scale rather than a sharp accessible/excluded boundary. With the nominal ζ input, BDC^2^^−^ (0.58) and BTC^3^^−^ (0.44) lie above this reference and form detectable shells, whereas DHTP^4^^−^ (0.33) lies slightly below 0.37 and does not form a detectable shell on t‐Cu_2_O (Table S2). The calculated depletion relative to the bulk is approximately 42%, 56%, and 67% for H_2_BDC, H_3_BTC, and H_4_DHTP, respectively. DHTP^4^^−^ nevertheless forms a thin shell on the weakly charged o‐Cu_2_O (ζ = −2.09 mV), for which the nominal accessibility is higher.

Because ζ is used as a surrogate input for ψ_0_, the absolute ratios and their positions relative to 1/e depend on the assumed interfacial potential. The ordering BDC^2^^−^ > BTC^3^^−^ > DHTP^4^^−^ is preserved for a fixed negative potential, but the location of any individual linker relative to the 1/e reference is not invariant. Moreover, the model does not consider finite ion size, double‐layer screening, linker speciation, specific adsorption, and surface coordination and is based on only three linkers. The calculation is therefore used as a physically motivated correlation between linker accessibility and shell formation rather than as a predictive criterion (Supporting Note S4).

Accordingly, within the proposed model, formation of the confining shell is favored when an anionic linker retains sufficient near‐surface accessibility despite electrostatic repulsion. Under the tested conditions, BTC^3^^−^ and BDC^2^^−^ showed sufficient accessibility to support shell formation, whereas DHTP^4^^−^ did not on t‐Cu_2_O. The ∼1/e reference is used only as a heuristic reference scale to organize these observations, rather than a predictive boundary. Because linker accessibility also depends on facet type, pH, and ionic strength, this correlation provides a rational basis for examining additional frameworks. The extension beyond HKUST‐1 is demonstrated at the level of shell formation and locally identified Cu–BDC‐like crystalline domains, while the bulk phase purity, yield, porosity, and general framework applicability require further investigation.

## 3 Conclusions

This study demonstrates a self‐assembled nanoreactor method that uses photoinduced release of Cu^2+^ from Cu_2_O NPs to generate localized supersaturation and form highly crystalline HKUST‐1 under mild aqueous conditions. We propose a mechanism in which shell formation establishes an *S*_meta_ regime, continued Cu release and restricted exchange drive the local environment toward *S*_labile_, and crystallization proceeds predominantly inward at or near the core–shell interface. This model integrates the measured Cu‐release response, morphology‐dependent shell formation, transport perturbations, shell composition, and reaction–diffusion analysis into a unified description of the crystallization process.

By tuning the interplay between *k*_elute_ and *D*_acc_, nucleation and growth can be spatially regulated, enabling control of the crystal morphology from dense, well‐faceted architectures to hollow structures, within a single synthetic platform. Furthermore, structural characterization confirmed the successful formation of highly crystalline HKUST‐1, highlighting the pivotal role of localized supersaturation in modulating nucleation and growth dynamics. Experiments on additional Cu‐MOF linkers show that BDC^2−^ forms shell‐bearing intermediates and local Cu–BDC‐like crystalline domains, whereas DHTP does not form a detectable shell on t‐Cu_2_O, identifying linker accessibility as a practical constraint on the current scope of the strategy.

More broadly, this approach demonstrates that crystallization can be programmed through the spatial and temporal localization of reactants rather than by uniformly increasing precursor concentration or thermal energy. Localized supersaturation therefore provides a reaction framework for controlling nucleation and crystal growth under mild conditions and offers a potential route toward the spatially programmed synthesis of functional crystalline materials.

## 4 Experimental Section

### 4.1 Materials

Copper(II) chloride (CuCl_2_, 97%, Sigma‐Aldrich), PVP (Mw ∼55,000, Sigma‐Aldrich), sodium hydroxide (97%, Sigma‐Aldrich), L‐ascorbic acid (≥99%, Sigma‐Aldrich), ethanol (≥99.5%, Sigma‐Aldrich), benzene‐1,3,5‐tricarboxylic acid (BTC, 95%, Sigma‐Aldrich), TEA (≥99%, Sigma‐Aldrich), and deionized water (DI water, 18.2 MΩ) were used throughout the experiments.

### 4.2 Synthesis of Cu_2_O Nanoparticles

To synthesize Cu_2_O NPs with cubic, truncated octahedral, and octahedral morphologies, PVP was added to DI water (7.0 mL) in amounts of 0, 0.3, and 1.0 g, respectively. Then, an aqueous CuCl_2_ solution (0.1 M, 1.0 mL) was added under stirring before the PVP was completely dissolved. The mixture was maintained at 55°C for 30 min. Subsequently, NaOH aqueous solution (2.0 M, 1.0 mL) was added dropwise (<1 min) under vigorous stirring and maintained at 55°C for 30 min. To this mixture, an aqueous ascorbic acid solution (0.6 M, 1.0 mL) was added dropwise (<1 min) and maintained at 55°C for 3 h. The obtained Cu_2_O NPs were centrifuged at 6,000 rpm for 10 min. The supernatant was discarded, and the precipitate was redispersed in DI water. A similar cleaning procedure was repeated three times, and the cleaned precipitate was redispersed in ethanol.

### 4.3 Preparation of H_3_BTC Solution

H_3_BTC (0.642 g), TEA (0.912 g), and DI water (10.0 mL) were vortexed until the H_3_BTC powder was dissolved to form a transparent solution.

### 4.4 Formation of HKUST‐1

First, t‐Cu_2_O NPs dispersed in ethanol were redispersed in deionized water. The NP solution (1.0 mL) and an H_3_BTC solution (1.0 mL) were combined in a 15 × 45 mm glass vial, which was placed on the self‐fabricated reaction holder 0.5 cm from a light source. The mixture was exposed to visible light at an intensity of 0.960 mW cm^−2^ at 25°C; under these conditions, light irradiation induced negligible heating of the solution. The irradiation time varied from 1 to 100 min. At the single‐particle level (on the optical microscope), the conversion of an individual Cu_2_O NP into a crystalline HKUST‐1 particle was completed in under 1 min; however, because the reaction‐initiation times of individual NPs are distributed, longer irradiation of the ensemble was required to obtain a practical yield. The product yield increased with irradiation time up to 100 min, which was therefore used as the standard reaction time for the nano‐HKUST‐1 product shown in Figure 4. Longer irradiation is not recommended beyond this point because the deprotonated framework began to undergo dissociation after approximately 2 h of continued illumination. After irradiation, the product was recovered by the following ethanol‐based extraction process, the mechanistic basis of which is detailed in Supporting Note S3.

### 4.5 Ethanol‐Based Extraction Process

The reaction solution (2.0 mL) was divided into two microtubes (1.0 mL each) and centrifuged at 10,000 rpm for 10 s. The supernatant was carefully decanted in a glass vial, and ethanol (1.0 mL) was added gently. The mixture gradually formed a blue layer at the interface. After phase separation, the upper layer containing the blue layer was collected and centrifuged at 10,000 rpm for 2 min. The cleaning procedure was repeated three times using ethanol. The acquired HKUST‐1 solution was dried under vacuum to obtain crystalline HKUST‐1.

## Author Contributions

**Yongdeok Ahn**: conceptualization, investigation, Writing – original draft, Writing – review and editing, visualization, validation, methodology, formal analysis, data curation, resources, software. **Seunghyeon Jeong**: writing – original draft, writing – review and editing, visualization, validation, methodology, formal analysis, data curation, investigation, resources, software. **Sun Ho Park**: methodology, validation, visualization, writing – review and editing, writing – original draft, formal analysis, data curation, investigation, resources. **Juhyeong Cho**: methodology, validation, visualization, writing – review and editing, resources. **Nak Cheon Jeong**: conceptualization, investigation, funding acquisition, writing – original draft, writing – review and editing, supervision, project administration. **Daeha Seo**: conceptualization, investigation, funding acquisition, writing – original draft, writing – review and editing, visualization, project administration, supervision, resources.

## Conflicts of Interest

The authors declare no conflicts of interest.

## Supporting information




**Supporting File 1**: adma75080‐sup‐0001‐SuppMat.docx.

**Supporting File 2**: adma75080‐sup‐0002‐MovieS1.mp4.

## Data Availability

The data supporting the results in this study are available within the paper and its Supporting Information. The raw and analyzed datasets generated during the study are available for research purposes from the corresponding author upon reasonable request.
