# Supplementary material for: Light‐Programmed Localized Supersaturation Enables Ambient Aqueous Synthesis of Highly Crystalline Metal–Organic Frameworks
Source: Adv Mater. 2026 Sep 18;38(62):e75080. doi: 10.1002/adma.75080 (PMC13741114; doi:10.1002/adma.75080)
Supplement: Supplementary file 1 [file ADMA-38-e75080-s002.docx]

Supporting Information

Light-Programmed Localized Supersaturation Enables Ambient Aqueous Synthesis of Highly Crystalline Metal–Organic Frameworks

Yongdeok Ahn,^1,2,3,#^ Seunghyeon Jeong^1, 2,#^ Sun Ho Park ^2,#^ Juhyeong Cho,^1,2^ Nak Cheon Jeong,^2,^* Daeha Seo^1,2,^*

^#^Y. Ahn, S. Jeong and S. H. Park contributed equally to this work.

*Correspondence should be addressed to N. C. J. ([nc@dgist.ac.kr](mailto:nc@dgist.ac.kr)), and D. S. ([daehaseo@postech.ac.kr](mailto:daehaseo@postech.ac.kr)).

Y. Ahn, S. Jeong, J. Cho, D. Seo

^1^Department of Chemistry, POSTECH

Pohang 37673, Republic of Korea

E-mail: [daehaseo@postech.ac.kr](mailto:daehaseo@postech.ac.kr)

Y. Ahn, S. Jeong, S. H. Park, J. Cho, N. C. Jeong, D. Seo

^2^Department of Physics and Chemistry, DGIST

Daegu 42988, Republic of Korea

E-mail: [nc@dgist.ac.kr](mailto:nc@dgist.ac.kr)

Y. Ahn

^3^Department of Chemistry Education, Gyeongsang National University

Jinju 52828, Republic of Korea

**Supporting Figures**

**Figure S1. Step-by-step schematic of the proposed strategy.** Upon light irradiation, eluted metal ions coordinate with ligands to form secondary building units (SBUs). As local enrichment reaches the metastable regime, a polycrystalline or porous shell forms on the nanoparticle surface. As enrichment increases further into the labile regime, interface-driven crystal growth proceeds predominantly inward at or near the core–shell interface, leading to well-crystallized materials.

**Figure S2. Schematic of supersaturation (*S*)-dependent nucleation and crystal growth.** a) Relationship between *S* and volume free energy (Δ*G*_v_) based on classical nucleation theory. b) Relationship between free energy (Δ*G*) and critical radius (*r*_c_). The red, green, and black curves represent *S*_high_, *S*_mid_, and *S*_low_ levels, respectively. c). Relationship between *r*_c_ and *S*.

**Figure S3. Structural and morphological characterization of shape-controlled Cu_2_O NPs.** By adjusting the concentration of PVP, Cu_2_O NPs with three different shapes were synthesized. c-Cu_2_O and o-Cu_2_O consist exclusively of (100) and (111) facets, respectively, whereas t-Cu_2_O incorporates both (100) and (111) facets. These distinct morphologies expose different terminal atomic configurations, which in turn influence their respective *k*_elute_ and *F*_rep_ values.

**Figure S4. Estimation of the band energy levels of Cu_2_O NPs with different shape.** a) Ultraviolet photoelectron spectroscopy (UPS) results and b) optical band gaps obtained from Tauc plots of c- (blue), t- (red), and o-Cu_2_O (yellow) NPs.

**Figure S5. Schematic of intraparticle charge transfer in the (100)/(111) facet junction.** Upon light irradiation, photoinduced charge carriers are generated both on the surface (bottom left) and within the interior (bottom right) of t-Cu_2_O. On the surface, electrons excited on the (100) facet migrate to the CB of the (111) facet, while holes generated on the (111) facet migrate to the VB of the (100) facet, achieving spatial charge across the surface interface. In parallel, within the core (interior), photogenerated electron–hole pairs separate along the asymmetrical band structure and transfer directionally toward the (111) and (100) facets, respectively. The interior region functions as both a source and mediator of charge carriers, enabling dual-pathway separation without an external electron mediator. The accumulation of photogenerated holes at the (100) facet enhances self-photooxidation activity, promoting Cu^2+^ elution.

**Figure S6. Facet-dependent self-photooxidation behaviors of Cu_2_O nanoparticles under visible-light irradiation.** a) Representative SEM images of o-Cu_2_O, c-Cu_2_O, and t-Cu_2_O nanoparticles before (top) and after (bottom) visible-light irradiation. All three morphologies undergo self-photooxidation upon illumination, accompanied by facet-dependent surface etching and morphological evolution. b) Relative concentration of eluted Cu species (*C*_eluted_/*C*_0_) measured after visible-light irradiation by ICP-MS. The amount of released Cu depends strongly on nanoparticle morphology, indicating facet-dependent self-photooxidation kinetics. Statistical significance was evaluated using one-way ANOVA with Tukey’s post-hoc test. *p < 0.05, **p < 0.01.

**Figure S7. XPS analysis of residual PVP on Cu_2_O nanoparticles with different morphologies.** a) Cu 2p_3/2_ spectra used for normalization. b) N 1s spectra originating from the pyrrolidone nitrogen of PVP. c) Relative N/Cu area ratio obtained by normalizing the N 1s peak area to the Cu 2p_3/2_ peak area. Although t-Cu_2_O and o-Cu_2_O were synthesized with substantially different PVP loadings, both exhibit similar residual PVP levels after washing. Together with the shell-formation behavior, these results indicate that residual PVP is not the dominant factor governing shell formation.

**Figure S8. Photographs of the laboratory-built reaction vessel for controlled photo-assisted synthesis.** Front, side, and top views show the 3D-printed holder that fixes the reaction vial in place, allows light irradiation from below using a mercury lamp, and maintains a constant distance between the light source and sample to ensure uniform exposure during the photo-assisted reaction.

**Figure S9. Time-dependent thermal monitoring during visible-light irradiation.** Representative thermal images of the reaction vessel recorded at 0, 1, and 100 min during visible-light irradiation. The measured temperature remained nearly constant throughout the reaction (25.5–25.6 °C), indicating that heat generation under the employed irradiation conditions was negligible and that the observed reaction cannot be attributed to a photothermal effect.

**Figure S10.** **FTIR analysis of core–shell nanoparticles.** FTIR spectra of the core–shell intermediate and pristine HKUST-1. The core–shell intermediate exhibits O–H stretching, carboxylate O–C=O stretching, C–O stretching, and Cu–O vibrational features comparable to those of pristine HKUST-1, indicating partial coordination of BTC ligands to Cu species on the nanoparticle surface. These results support the formation of a Cu–BTC-containing shell before full crystallization into HKUST-1.

**Figure S11. XPS analysis of Path A (core–shell intermediate) and Path B (stirred product).** a) High-resolution C 1s, O 1s, and Cu 2p_3/2_ XPS spectra of the core–shell intermediate (Path A). The C 1s spectrum contains a distinct carboxylate (COO^⁻^) contribution, indicating BTC coordination at the shell surface. The O 1s spectrum contains contributions from Cu–O, Cu–OH, and carboxylate/C–O species. The Cu 2p_3/2_ spectrum is dominated by Cu^+^ with only a minor Cu^2+^ contribution, consistent with a Cu^+^-rich, partially coordinated shell surface. b) High-resolution C 1s, O 1s, and Cu 2p_3/2_ XPS spectra of the product obtained under stirring (Path B). Compared with the core–shell intermediate, the stirred product exhibits a lower carboxylate-to-aliphatic carbon ratio in the C 1s spectrum, indicating a lower extent of BTC coordination at the surface. In contrast, the O 1s and Cu 2p_3/2_ spectral envelopes remain largely similar, suggesting that the primary difference between the two pathways arises from the degree of metal–linker coordination rather than the overall oxygen or copper chemical environment.

**Figure S12. HR-TEM and FFT analysis of the core–shell intermediate.** a, b) Representative HR-TEM images acquired from crystalline regions of the shell along with the corresponding FFT patterns. Discrete diffraction spots and rings are observed in the FFT patterns, and the measured interplanar spacings are consistent with Cu(OH)_2_, confirming the presence of locally dispersed crystalline Cu(Ⅱ) hydroxide domains within the shell. Residual Cu_2_O lattice reflections are also observed in some regions. c, d) Representative HR-TEM images acquired from other shell regions and their corresponding FFT patterns. The absence of discrete diffraction features and the presence of diffuse FFT halos indicate predominantly amorphous, non-crystalline material lacking long-range order. The coexistence of crystalline and amorphous domains indicates that the shell is structurally heterogeneous and is not well described as a single crystallographic phase.

**Figure S13. Raman spectroscopic comparison of Path A (stationary) and Path B (stirred) products.** a) Raman spectra obtained during the confined crystallization pathway (Path A). Compared with pristine Cu_2_O nanoparticles, spectra collected after 30 and 60 min show the emergence of BTC-related vibrational modes, including aromatic C–H, C=C, and carboxylate (COO^-^) bands, along with the attenuation of Cu_2_O features, indicating the progressive formation of Cu–BTC coordination species during shell evolution. b) Raman spectra collected from spatially distinct regions of the stirred product (Path B). Different positions exhibit different spectral signatures, indicating spatially heterogeneous coordination environments. In Position 1, carboxylate (1447 and 1589 cm⁻¹), aromatic C–H (731 and 816 cm⁻¹), and C=C (1008 cm⁻¹) modes are consistent with Cu–BTC coordination; however, their frequencies differ from those of HKUST-1, indicating that stirring opens multiple coordination pathways rather than selectively directing framework formation toward HKUST-1.

**Figure S14. Suppression of Cu release into the bulk solution by shell formation.** Cu concentration in the bulk solution measured by ICP-OES during the core–shell formation stage with and without BTC ligands. Without ligands, a substantial amount of Cu is released into the bulk solution, whereas the presence of ligands markedly decreases the detected bulk Cu concentration. This reduction suggests the formation of a ligand-induced shell, which restricts outward Cu transport and retains released Cu species near the NP surface. Because ICP-OES quantifies dissolved Cu rather than providing a complete Cu mass balance, precipitation, adsorption, or complexation may also contribute to the detected Cu level. These data therefore support a transport-restricting role for the shell rather than establishing it as a near-permeable barrier.

**Figure S15. External metal-cation tracer experiment after shell formation.** TEM/STEM images and corresponding EDS elemental maps of the crystals obtained after adding external a) Zn^2+^ and b) Mn^2+^ cations to the reaction solution following core–shell formation but before the completion of crystallization. In both cases, the final crystals show a strong Cu signal, whereas Zn and Mn signals are not detected within the particles at the sensitivity of EDS elemental mapping. This indicates that incorporation of the externally added metal cations into the growing framework, if present, is below the detection level of this measurement, consistent with restricted inward metal-ion transport across the shell during crystallization. However, without appropriate positive controls, quantitative detection limits, and bulk compositional analysis, the experiment cannot exclude low-level cation ingress.

**Figure S16. External linker tracer experiment after shell formation.** Representative SEM images of the final products obtained after introducing structurally distinct linkers following core–shell formation but before the completion of crystallization: a) BDC and b) DHTP. Despite the addition of linkers that normally direct the formation of Cu-MOF-2 and Cu-MOF-74, the resulting particles retained the characteristic octahedral morphology and size of HKUST-1. This indicates that the externally added linkers did not noticeably alter the particle morphology, which is consistent with their limited participation in framework assembly and confirms the transport-restricting role of the shell. However, morphology alone does not exclude low-level linker incorporation, which would require compositional analysis of the digested product.

**Figure S17. Formation of nano-octahedral HKUST-1 crystals after light irradiation.** a) TEM and b) SEM images of the final products obtained after further visible-light irradiation of the core–shell intermediates. The particles exhibit well-defined nano-octahedral morphology characteristic of HKUST-1, indicating that continued light-driven self-photooxidation converts the core–shell intermediates into crystalline nano-HKUST-1.

**Figure S18. Schematic of the ethanol-based extraction process for collecting nano-HKUST-1 crystals.** Unreacted Cu_2_O nanoparticles were first removed from the reaction mixture by centrifugation. Ethanol was then layered onto the aqueous supernatant to quench framework evolution and induce the interfacial precipitation of HKUST-1. This ethanol-based extraction process enables the efficient recovery of highly crystalline nano-octahedral HKUST-1 products under mild conditions. A detailed mechanistic interpretation is provided in Supporting Note 3.

**Figure S19. Control experiment for ethanol-based extraction in the absence of light-driven synthesis.** SEM image of the product obtained by applying the same ethanol-based extraction process to a solution containing Cu^2+^, H_3_BTC, and TEA at the same concentrations as the post-reaction mixture, but without visible-light irradiation. No well-defined octahedral HKUST-1 crystals were formed; instead, non-identified slurry-like aggregates were observed. This result shows that ethanol-based extraction alone does not generate crystalline HKUST-1 or its nano-octahedral morphology, indicating that the observed HKUST-1 crystals originate from the light-driven synthesis rather than from the extraction step.

**Figure S20. Control experiments using physical isolation and acid quenching.** a) SEM image of HKUST-1 crystals recovered by purely physical isolation using an Amicon ultrafiltration membrane, without chemical quenching. The recovery of octahedral HKUST-1 confirms its formation during the light-driven synthesis before ethanol-based extraction. b) SEM image of the product obtained after quenching with acetic acid. Only fragmented and disrupted material is observed, indicating that abrupt bulk-acid protonation destabilizes the framework rather than recovering intact HKUST-1. These control experiments show that ethanol-based extraction does not create the HKUST-1 morphology, but instead stabilizes and recovers pre-formed crystals through controlled interfacial protonation.

**Figure S21. Comparison of the size distributions of t-Cu_2_O nanoparticles and HKUST-1 crystals.** a) Size distribution of the t-Cu_2_O nanoparticles showing an average edge length of 239.0 ± 34.6 nm. b) Size distribution of the final nano-HKUST-1 crystals showing an average edge length of 242.4 ± 35.7 nm. c) Statistical comparison of particle edge lengths. No significant difference is observed between the two populations (unpaired two-tailed Student’s t-test, p = 0.1234, ns; not significant). The nearly identical mean sizes and overlapping distributions indicate that the dimensions of the final HKUST-1 crystals correspond closely to those of the parent Cu_2_O NPs, supporting particle-templated, predominantly inward growth within the shell defined by the original NP.

**Figure S22. Light intensity-dependent Cu^2+^ elution kinetics used to control *k*_elute_.** a) Linear relationship between incident light intensity and the Cu^2+^ elution rate constant, *k*_elute_, obtained from single-particle dark-field scattering measurements. The strong linear correlation (*R*^2^ = 0.999) indicates that Cu_2_O self-photooxidation can be quantitatively controlled by light intensity. b) Simulated Cu^2+^ elution rate profiles from individual t-Cu_2_O nanoparticles under different light intensities. The geometric and Rayleigh models estimate the time-dependent Cu^2+^ release behavior, showing that decreasing the light intensity lowers the initial elution rate and broadens the Cu^2+^ release window. This tunability of *k*_elute_ provides a basis for modulating crystallization kinetics and promoting hollow HKUST-1 formation.

**Figure S23. Morphological outcomes obtained by the kinetic modulation of the crystallization process.** a) SEM image of the product obtained under pulsed-light irradiation (5 s light-on/5 s light-off cycles, total irradiation time 1 min). The product consists of aggregated nanoparticle-like structures rather than uniform hollow HKUST-1. b) SEM image of the product synthesized at a reduced temperature (3 °C). Lowering the reaction temperature yielded plate-like structures instead of the characteristic octahedral HKUST-1 morphology. These results indicate that neither periodic interruption of Cu^2+^ release nor uniform kinetic slowdown is sufficient to achieve the formation of uniform hollow HKUST-1, highlighting the need for a synchronized and spatially uniform modulation of *k*_elute_ diffusion and crystallization kinetics across the nanoparticle ensemble.

**Figure S24. Ex situ TEM analysis of the crystallization time scale.** Representative TEM images of independently prepared samples collected at different irradiation times during the transformation of t-Cu_2_O nanoparticles into HKUST-1. The octahedral morphology is already established after 1 min of irradiation and remains largely unchanged throughout the subsequent reaction period, indicating that conversion is substantially complete within approximately 1 min at the ensemble level. Because each image captures a different population of particles rather than following the same particle continuously, these images establish the timescale of the transformation at the ensemble level but do not constitute a continuous observation of inward crystallization within a single particle. Nevertheless, extended irradiation is required at the ensemble level to increase product yield because individual nanoparticles initiate the transformation at different times. The particle morphology remains intact for up to 100 min, whereas prolonged irradiation (24 h) leads to framework degradation.

**Figure S25. FTIR analysis of the final HKUST-1 product.** FTIR spectra of the final product obtained in this study and pristine HKUST-1. The final product exhibits characteristic asymmetric carboxylate stretching bands in the 1600–1500 cm^-1^ region, benzene-ring vibration bands in the 1400–1300 cm^-1^ region, and Cu–O stretching bands at 700–750 cm^-1^, all of which closely match those of pristine HKUST-1. This spectral agreement confirms the successful formation of the HKUST-1 framework through light-driven synthesis.

**Figure S26. Raman spectroscopic analysis of products obtained with BDC and DHTP linkers.** a) Raman spectra of the BDC system collected from the core–shell intermediate and the extracted final product. The core–shell intermediate exhibits characteristic bands of both the Cu_2_O core (145, 219, 405, and 626 cm^-1^) and coordinated BDC (864, 1137, 1433, and 1609 cm^-1^), whereas the extracted product displays bands consistent with the reported spectra of crystalline Cu–BDC (Cu-MOF-2; 866, 1142, 1449, and 1614 cm^-1^), suggesting local Cu–BDC-like crystalline domains in the material examined. Raman spectroscopy analysis is performed on selected regions of the product and does not establish the bulk phase identity, including phase fraction, purity, yield, and porosity of the recovered solid. b) Raman spectra of the DHTP system. Although Cu–carboxylate coordination is present, the characteristic C–O stretching band of crystalline Cu-MOF-74 near 1273 cm^-1^ is not observed. Along with the absence of a core–shell intermediate in HR-TEM analysis, these results indicate that DHTP does not form a detectable shell on the nanoparticle surface under the present conditions.

**Figure S27. HR-TEM/FFT analysis of BDC-derived core–shell intermediates and BDC-derived crystalline domain.** a) HR-TEM image and corresponding FFT pattern of the BDC-derived core–shell intermediate. The shell region exhibits predominantly amorphous characteristics with no well-defined long-range periodicity, consistent with the formation of a metastable intermediate before framework crystallization. b) HR-TEM image and corresponding FFT pattern of the final BDC-derived product, in which the diffraction features are consistent with those of Cu–BDC, demonstrating the formation of Cu–BDC-like crystalline domains. As a single-particle probe, HR-TEM/FFT does not provide information on the bulk phase identity, including phase fraction and porosity of the recovered product.

**Figure S28. HR-TEM/FFT analysis of DHTP-derived products on t-Cu_2_O and o-Cu_2_O.** a) HR-TEM image and corresponding FFT pattern of t-Cu_2_O reacted with DHTP under fully deprotonating conditions. No core–shell intermediate is observed, and the FFT pattern is dominated by the reflections of Cu_2_O lattice, indicating that DHTP does not nucleate on the strongly negatively charged t-Cu_2_O surface. b) HR-TEM image and corresponding FFT pattern of o-Cu₂O reacted with DHTP. In contrast to t-Cu_2_O, a thin shell is observed on the weakly charged o-Cu_2_O surface. This observation is consistent with the higher nominal electrostatic accessibility of DHTP at lower surface potential, supporting electrostatic accessibility as an important contributing factor in shell formation rather than an independently validated criterion.

**Table S1. Synthesis conditions, surface properties, and shell-formation outcomes for the three Cu_2_O NP morphologies.** PVP was used at three loadings as a morphology-directing agent rather than a surface stabilizer. Residual PVP, quantified by XPS as the N 1s/Cu 2p_3/2_ area ratio normalized to c-Cu_2_O, remains at a near-saturated level and does not increase with loading. The PVP-free c-Cu_2_O forms no shell, and the two PVP-bearing samples with nearly identical residual PVP give different morphology outcomes, indicating that electrostatic repulsion (as reflected in the measured zeta potential), rather than residual PVP, is the major factor governing shell formation under these conditions.

| **Parameter** | **c-Cu_2_O** | **t-Cu_2_O** | **o-Cu_2_O** |
| --- | --- | --- | --- |
| **Morphology** | Cubic | Truncated octahedral | Octahedral |
| **PVP loading (g)** | 0 | 0.3 | 1.0 |
| **Relative residual PVP**  **(N-to-C ratio)** | 1 | 3.5 | 3.7 |
| **Zeta potential (mV)** | −26.1 | −7.05 | −2.09 |
| **Shell formation** | None | Robust shell | Thin and partial shell |

**Table S2. Electrostatic accessibility of the three Cu-MOF linkers at the t-Cu_2_O surface.** The near-surface concentration of a fully deprotonated linker of charge *z* is reduced relative to the bulk by the Boltzmann factor *C*_surf_/*C*_bulk_ = exp(-*ze*ψ_0_/*kT*) with ψ_0_ ≈ ζ and *W*(*kT)/e* = 25.7 mV at 25 °C. The measured zeta potential ζ is used only as a nominal electrokinetic input to estimate $\psi_{0}$. Because ζ is defined at the slipping plane and $\left| \zeta\right|\leq\left| \psi_{0} \right|$, it is not equivalent to the true surface potential. Accordingly, the tabulated ratios are model-derived rather than measured near-surface concentration. The exclusion energy *W* = |*z*|*e*ψ_0_/*kT* equals one *kT* at *C*_surf_/*C*_bulk_ = 1/*e*, which is used here as a characteristic reference rather than a predictive boundary. With this nominal input, the *C*_surf_/*C*_bulk_ ratios of BDC^2−^ and BTC^3−^ lie above this reference and are associated with detectable shell formation, whereas the ratio for highly charged DHTP^4−^ lies slightly below the reference, corresponding to no formation of a detectable shell on t-Cu_2_O. The above mean-field treatment does not consider finite ion size, double-layer screening, linker speciation, and specific adsorption and is based on only three linkers.

| Linker | Charge | Framework | *kT* | *C*_surf_/*C*_bulk_ | Surface depletion |
| --- | --- | --- | --- | --- | --- |
| BDC | 2 | Cu-MOF-2 | 0.55 | 0.58 | 42% |
| BTC | 3 | HKUST-1 | 0.82 | 0.44 | 56% |
| DHTP | 4 | Cu-MOF-74 | 1.10 | 0.33 | 67% |

**Supporting Note 1. Estimation of the hydrodynamic force induced by stirring**

Hydrodynamic forces generated by magnetic stirring in a 2 mL vial were estimated to analyze their influence on fluid dynamics. The rotational motion of the magnetic stir bar and its effect on the surrounding fluid were quantified using classical mechanics and fluid dynamic principles. Fluids undergoing circular motion due to stirring experience a centripetal force, calculated as:

$$F=ma=m\times\frac{v^{2}}{r}$$

where *v* is the tangential velocity, *r* is the radial distance from the center of rotation, and *m* is the mass of the fluid. The equation for *v* is:

$v=r\cdot\omega$,

where ω is the angular velocity, defined as:

$$\omega=\frac{2\pi N}{60}$$

and *N* is the stirring speed in revolutions per minute (rpm).

To assess the flow regime, the Reynold number (*Re*) was calculated:

$Re=\frac{\rho vd}{\mu}$,

where *ρ* is the fluid density, *d* is the characteristic length (vial diameter), and *μ* is the dynamic velocity. The flow classifications based on *Re* are as follows:

- Laminar flow: *Re* < 2300
- Transitional flow: 2300 < *Re* < 4000
- Turbulent flow: 4000 < *Re*

For a 2 mL vial (*d* = 10 mm) containing water at 25°C (*ρ* = 997 kg m^-3^, *μ* = 0.89 × 10^-3^ Pa) stirred at 700 rpm with an estimated radial distance *r* = 5 mm, its *v* is expressed as

$$v=0.005\times\frac{2\pi\times700}{60}=0.366 ms^{-1}$$

The centripetal acceleration is:

$$a=\frac{v^{2}}{r}=\frac{\left( 0.366 \right)^{2}}{0.005}=26.79 ms^{-2}$$

Given the mass of the fluid:

$$m=\rho\times V=997\times2\times{10}^{-6}=0.001994 \mathrm{kg}$$

the force is:

$F=0.001994\times26.79=0.0534 N$.

Then, *Re* is calculated as:

$Re=\frac{997\times0.366\times0.01}{0.89\times{10}^{-3}}\approx4101$.

Since the calculated *Re* value exceeds the threshold for turbulence, the fluid flow is expected to be fully turbulent. In laminar flow, mixing is dominated by molecular diffusion, leading to slower and less homogeneous reactant dispersion. In contrast, turbulent flow enhances convective transport and facilitates rapid homogenization of the reaction medium, reducing the persistence of spatial concentration gradients.

**Supporting Note 2. Limiting-case estimation of the local Cu inventory around photoactive Cu_2_O nanoparticles**

The direct measurement of the Cu^2+^ concentration within the sub-micrometer region surrounding individual Cu_2_O nanoparticles (NPs) lies beyond the spatial and temporal resolution of currently available in situ analytical techniques. Therefore, all concentrations reported below are obtained under idealized assumptions (negligible leakage and consumption, with the solubility of simple Cu(II) salts as an approximate ceiling). The limitations of these assumptions are set out in Section 2.6. To address this limitation we constructed a limiting-case framework based on two experimental observations and reaction-diffusion analysis. The framework aims to provide an upper bound on the local Cu inventory rather than to predict its actual value:

(i) *Mass balance.* Inductively coupled plasma-optical emission spectroscopy (ICP-OES) measurements show that the dissolved Cu^2+^ concentration in the bulk during and after the core–shell formation stage is substantially below the value expected for complete dissolution of Cu_2_O into the bulk volume, implying that the released Cu^2+^ is retained near the NPs (**Figure S14**). This measurement reflects the concentration of dissolved Cu before a complete mass balance is established, considering that precipitation, adsorption, or complexation may also affect the results.

(ii) *Restricted transport across the shell.* External metal ions (e.g. Zn^2+^, Mn^2+^) introduced after shell formation are not detected by EDS in the HKUST-1 after subsequent crystallization **(Figure S15**, see also Section 2.2 below). This is consistent with restricted inward transport of metal cations across the shell. However, without positive incorporation control or a quantitative detection limit for EDS, this result does not provide conclusive evidence for a near-impermeable barrier.

Together, the above observations indicate strongly restricted exchange between the shell-bounded region and the bulk. Based on this assumption, we adopt a closed mass-balance limit as an idealized limiting case. Within this limiting case, the local Cu inventory is determined by the geometry of the cavity and an assumed solubility ceiling, rather than by Fickian leakage. All values obtained in this way represent upper bounds and should not be interpreted as measured local concentration or free-Cu^2+^ activities related to thermodynamic supersaturation. The spatiotemporal concentration profiles obtained from the reaction diffusion analysis are shown in **Movie S1**.

**2.1. Definition of reference concentrations**

The supersaturation ratio defined as *S* = [Cu^2+^]_local_/[Cu^2+^]_sat_ is the conventional metric for nucleation analysis. However, HKUST-1 is a coordination polymer formed by the reaction 3Cu^2+^ +2BTC^3-^ → Cu_3_(BTC)_2_, and the equilibrium concentration [Cu^2+^]_eq_ is determined by [BTC^3-^] through the solubility product *K*_sp_ = [Cu^2+^]_eq_^3^[BTC^3-^]^2^. Reliable *K*_sp_ values for HKUST-1 in the aqueous-TEA conditions used here have not been established in the literature, and reported solubilities for HKUST-1 in pure water span several orders of magnitude. Therefore, adopting any specific value of [Cu^2+^]_sat_ would introduce an arbitrary scaling that obscures the robustness of the underlying analysis. For reference, reported aqueous solubilities of Cu-trimesate species and the metastable-zone widths typical of carboxylate frameworks place the relevant nucleation and growth concentration broadly in the sub-mM to few-mM range. We use this range only as an order-of-magnitude reference for the apparent boundary *C*_critical_ between the *S*_meta_ and *S*_labile_ regimes, not as a measured threshold for the present system. All conclusions drawn below rely on relative local-to-bulk enrichment, which is independent of the poorly constrained *K*_sp_ and *C*_critical_.

To avoid ambiguity, the analysis below is framed in terms of relative enrichment ratios rather than absolute *S* values. The three key ratios are as follows.

(i) Local-to-bulk enrichment: [Cu^2+^]_local_/[Cu^2+^]_bulk_ is the spatial concentration contrast around an individual NP;

(ii) Pre-shell-to-post-shell amplification: The [Cu^2+^]_local_ increases upon shell formation;

(iii) Cavity-to-conventional ratio: [Cu^2+^]_cavity_/[Cu^2+^]_hydrothermal_ is the driving-force advantage over conventional hydrothermal synthesis, where *K*_sp_ cancels.

Each of these ratios is independent of the absolute *K*_sp_ value, making the analysis robust to *C*_sat_ uncertainty. Therefore, the regime labels *S*_meta_ and *S*_labile_ used in the main text are operational descriptors of the observed sequence (metastable: nucleation kinetically suppressed; labile: nucleation kinetically accessible), rather than quantitatively defined thermodynamic states or specific numerical thresholds.

**2.2. Experimental evidence for shell-mediated confinement**

**2.2.1 ICP-OES mass balance during and after core–shell formation**

ICP-OES measurements revealed that, within the timeframe relevant to core–shell formation, only a small fraction of the total Cu released from the parent t-Cu_2_O NPs is detected in the bulk solution. Given the NP loading used in our synthesis, the total Cu inventory upon complete dissolution would yield a bulk concentration in the millimolar range; the actual ICP-OES readings during and after the shell-formation phase are well below this expected concentration, implying that the released Cu^2+^ is not freely dispersed throughout the bulk volume but remains sequestered within the immediate vicinity of the NPs.

**2.2.2 Absence of post-shell metal ion intercalation**

A direct test of shell permeability was performed by adding external metal ions (Zn^2+^ and Mn^2+^) to the reaction solution after the core–shell intermediates were formed but before inward crystallization was complete. If the shell were permeable, these external cations would intercalate into the growing HKUST-1 framework, producing a mixed-metal product detectable by elemental analysis, such as energy dispersive X-ray spectroscopy (EDS). Instead, the resulting crystals were found to contain only Cu, with no incorporation of the external metal cations detectable by EDS (**Figure S15**). This result suggests restricted inward transport of metal cations across the shell. However, because this is a negative observation, a positive control demonstrating the expected incorporation in a fully accessible growing framework would be necessary to establish the sensitivity of the experiment and assess whether low-level cation ingress can be excluded.

Taken together, the observations detailed in sections 2.2.1 and 2.2.2 are consistent with the post-shell cavity operates in a regime in which Cu^2+^ leakage out of the cavity being substantially restricted on the timescale of crystallization, although they do not quantify the residual permeability. Based on this, we adopt a closed mass-balance system as a limiting idealization in the following analysis, and the resulting concentrations should be interpreted as upper bounds.

**2.3. Theoretical estimation of Cu^2+^ concentration under various conditions**

*Stage 1. Pre-shell open-boundary diffusion*

Before shell formation, Cu^2+^ released from the NP surface diffuses outward into a quiescent aqueous medium. Assuming spherical symmetry, the steady-state diffusion equation with a constant-flux Neumann boundary at the NP surface and a zero-concentration Dirichlet boundary at infinity yields:

| $C\left( r \right)=J\cdot\frac{{r_{NP}}^{2}}{D\cdot r}$ |  |
| --- | --- |
| $C\left( r_{NP} \right)=J\cdot\frac{r_{NP}}{D}$ | (S3-1) |

where *J* is the surface flux of Cu^2+^ generated by self-photooxidation, *r*_NP_ is the effective NP radius, *D* is the Cu^2+^ diffusion coefficient in water, and *r* is the radial distance from the NP center (*r ≥* *r*_NP_). The first equation gives the radial profile decaying as 1/*r*; the second is the steady-state value at *r* = *r*_NP_. Parameter values are listed in Table S3.^[1-7]^

**Table S3. Parameter values used in Eqs. S3-1 to S3-7, with literature and experimental sources.**

| Parameter | Value | Source and justification |
| --- | --- | --- |
| $\boldsymbol{r}_{\boldsymbol{NP}}$ | ~120 nm | Characteristic radius approximated as half the mean edge length of t-Cu_2_O NPs (239 ± 34.6 nm, *n* = 71; Figure S21a). For an order-of-magnitude estimate of diffusion from a faceted particle, this characteristic length suffices. The volume-equivalent sphere radius of an ideal truncated octahedron with the same edge length is larger by a factor of ~2.8; using the latter scales all estimated concentrations upward by a similar factor, preserving the qualitative conclusions of this analysis. |
| $\boldsymbol{\rho}_{\boldsymbol{Cu}_{\boldsymbol{2}}\boldsymbol{O}}$ | 6.0 × 10^3^ kg·m^-3^ | Crystallographic density of cuprite (Cu_2_O, space group $\boldsymbol{P}\bar{\boldsymbol{n}}\boldsymbol{3}\boldsymbol{m}$, *a* = 4.2696 Å). Calculated from the unit-cell parameters reported in the literature. |
| $\boldsymbol{M}_{\boldsymbol{Cu}_{\boldsymbol{2}}\boldsymbol{O}}$ | 143.09 × 10^-3^ kg·mol^-1^ | Molar mass of Cu_2_O calculated from IUPAC standard atomic weights (Cu: 63.546 g·mol^-1^, O: 15.999 g·mol^-1^). |
| $\boldsymbol{D}_{\boldsymbol{Cu}^{\boldsymbol{2+}}}$ | 7.1 × 10^-10^ m^2^·s^-1^ | Cu^2+^ tracer diffusion coefficient in dilute aqueous solution at 25 °C. Nernst–Hartley analysis of limiting ionic conductivities in the reported literature was used. |
| $\boldsymbol{t}_{\boldsymbol{complete}}$ | ~60 s at 0.96 mW·cm^-2^ | Single-particle photooxidation time of t-Cu_2_O under 0.96 mW·cm^-2^ white-light irradiation. Determined experimentally from the decay of dark-field single-particle scattering intensity (Figure S22, primary kinetic measurement), with ex situ TEM showing complete transformation (Figure S24). Light intensity was calibrated using a power meter and photodiode sensor. Linear scaling of the elution rate with light intensity over the range 0.03-0.96 mW·cm^-2^ was confirmed by ICP-MS (Figure S6b). |
| $\boldsymbol{\delta}$ | ~30 nm | Shell thickness, estimated from the TEM images of core–shell intermediates (Figure 3a) |

The total Cu inventory in a single NP and the ensemble-averaged surface flux are:

| $n_{\mathrm{Cu}}=2\times\left( \frac{4}{3} \right)\pi{r_{\mathrm{NP}}}^{3}\cdot\frac{\rho}{M}=6.07\times{10}^{-16} \mathrm{mol}$ | (S3-2) |
| --- | --- |
| $J_{\mathrm{avg}}=\frac{\left( \frac{n_{\mathrm{Cu}}}{t_{\mathrm{complete}}} \right)}{\left( 4\pi{r_{\mathrm{NP}}}^{2} \right)}\approx5.6\times{10}^{-5}mol\cdot m^{-2}\cdot s^{-1}$ | (S3-3) |

where *n*_Cu_ is the total Cu inventory per NP (factor of 2 from Cu_2_O stoichiometry), and π*r*_NP_^2^ is the spherical surface area. Substituting J_avg_, r_NP_, and D (Table 3) into Eq. S3-1 yields a steady-state surface concentration of approximately 9.5 μM above the bulk Cu^2+^ level. Single-particle scattering measurements (**Figure 2f**) reveal that the photodecomposition exhibits a burst regime with a characteristic half-life of 5–10 s, during which the instantaneous flux is ~8 times the ensemble average. This yields a peak surface concentration of approximately 76 μM during the burst.

Therefore, the local-to-bulk enrichment ratio at the pre-shell NP surface is approximately 8–80 relative to the bulk Cu^2+^ concentration during active photodecomposition. Although this represents meaningful local enrichment, the absolute concentration on the order of tens-of-micromolar is modest compared to the millimolar levels typically required for spontaneous HKUST-1 nucleation under conventional conditions. Therefore, the pre-shell stage alone is insufficient to drive rapid crystallization, consistent with the experimental observation that crystal formation occurs only after the shell has formed (Section 2.5 of the manuscript).

*Stage 2. Post-shell closed-cavity accumulation*

Combining the two experimental observations described in Section 2.2 (negligible ICP-OES leakage and absence of post-shell ion intercalation), we treat the post-shell cavity as a closed mass-balance system in which Cu^2+^ released from the receding core accumulates within the cavity volume bounded by the shell. The general mass balance for the free Cu^2+^ concentration in the cavity is:

| $\frac{dC_{cavity}}{dt}=J\cdot\frac{A_{core}}{V_{cavity}}-\frac{\frac{D_{shell}}{\delta^{2}}}{C_{cavity}-C_{bulkt}}-k_{form}\left[ {Cu}^{2+} \right]\left[ {BTC}^{3-} \right]$ | (S3-4a) |
| --- | --- |

where the three terms on the right-hand side represent (i) Cu^2+^ generation by the receding core, (ii) leakage through the shell, and (iii) consumption by SBU formation. The experimental results (**Figures S14 and S15**) indicate that the leakage term is small; we accordingly set *D*_shell_ → 0 as a limiting idealization. To estimate the pre-nucleation driving force—the maximum Cu^2+^ concentration available to drive nucleation—we set the consumption term to zero; this simplification is justified in detail in the following section. With both sink terms removed, the cavity concentration is given simply by the released inventory divided by the cavity volume:

| $C_{cavity}\left( t \right)=\frac{n_{Cu, released}\left( t \right)}{V_{cavity}\left( t \right)}$ | (S3-4b) |
| --- | --- |

where *n*_Cu,released_(*t*) is the cumulative amount of Cu^2+^ released from the receding core up to time *t*, and *V*_cavity_(*t*) is the corresponding cavity volume. Therefore, the concentration gradient in Eq. S3-4a is an upper-bound driving force, not the actual concentration at a steady state.

*Theoretical upper bound from solid-state Cu density*

The absolute upper bound from the solid-state Cu density in solid Cu_2_O, which represents the density before any volume expansion, is expressed as follows:

| $\left[ \mathrm{Cu} \right]_{\mathrm{solid}\mathrm{Cu}_{2}O}=\frac{2\rho}{M}=\frac{2\times6.0\times{10}^{3}}{143.09\times{10}^{-3}}=83.9 mol\cdot L^{-1}\approx84 M$ | (S3-5) |
| --- | --- |

This represents the concentration of Cu atoms within the Cu_2_O crystal lattice. No process that confines released Cu^2+^ within a finite volume can exceed this density.

*Geometric upper bound for the shell-bounded cavity*

In the limit of complete core dissolution (*r*_core_ → 0), the cavity occupies the volume of the entire shell-bounded region $V_{cavity,max}=\left( \frac{4}{3} \right)\pi\left( r_{\mathrm{NP}}+\delta\right)^{3}$, with *r*_NP_ = 120 nm and δ =30 nm:

| $V_{cavity,max}=\left( \frac{4}{3} \right)\pi\left( 150\times{10}^{-9} \right)^{3}=1.41\times{10}^{-20} m^{3}=1.41\times{10}^{-17} L$ | (S3-6) |
| --- | --- |
| $\left[ {Cu}^{2+} \right]_{cavity,max}=\frac{n_{Cu}}{V_{cavity,max}}=\frac{6.07\times{10}^{-16}}{1.41\times{10}^{-17}}\approx43 M$ | (S3-7) |

Thus, under the limiting assumption that all released Cu^2+^ remain confined within the shell-bounded cavity, the nominal geometric bound is approximately ~43 M, or about half the solid-state Cu density (Eq. S3-5). This is a mass-balance construct corresponding to perfect confinement; it is neither a realizable nor experimentally measured concentration.

*Practical limit from Cu^2+^ salt solubility*

The geometric upper bound of 43 M cannot be physically realized because it would require Cu^2+^ concentrations exceeding the solubility limits of all known aqueous Cu(Ⅱ) salts. The relevant solubility ceilings at 25 °C are controlled by

- Solid-state Cu in Cu_2_O: 84 M (lattice density, theoretical absolute limit)
- Geometric cavity limit under perfect barrier: 43 M
- Pure water (solvent capacity reference): 55M
- CuCl_2_ saturation in water: ~5.6 M^[8]^
- Cu(NO_3_)_2_ saturation in water: ~4.4 M^[9]^

If the cavity Cu^2+^ concentration were to approach the Cu(Ⅱ) salt solubility limit (~5 M, taken as an order-of-magnitude proxy; see Section 2.6),^[10,11]^ continued Cu^2+^ generation by the photoactive core would be balanced by the precipitation of secondary phases, including hydroxide and partially coordinated Cu-carboxylate intermediates. This is consistent with the high-resolution TEM/FFT indexing of crystalline domains to Cu(OH)_2_, the intermediate Raman signature in **Figure 3e**, and the ultimate SBU formation upon BTC^3-^ availability. Therefore, within this limiting calculation, the external ceiling on the cavity Cu^2+^ inventory is taken to be on the order of a few molar (~5 M). This value is an assumed order-of-magnitude proxy imposed on the calculation rather than a measured or predicted concentration for the Cu_2_O/H_3_BTC/TEA system, in which hydrolysis, complexation, Cu(OH)_2_ precipitation, and Cu-carboxylate formation govern the free-Cu^2+^ activity.

*Time scale to reach the solubility ceiling*

The rate of Cu^2+^ accumulation within the cavity in the closed-system limit is:

| $\frac{dC_{\mathrm{cavity}}}{dt}=\frac{J\cdot A_{\mathrm{core}}\left( t \right)}{V_{\mathrm{cavity}}\left( t \right)}$ | (S3-8) |
| --- | --- |

Immediately after shell formation, *A*_core_ = *A*_NP_ and *V*_cavity_ is small (a thin spherical shell). The volumetric accumulation rate is approximately *J*·*A*_NP_/*V*_cavity,initial_, giving ~60 mM·s^-1^ (ensemble flux) or ~480 mM·s^-1^ (burst flux) at the just-formed shell. Under the limiting assumption of negligible leakage and consumption, integrating the burst duration of ~10 s gives an accumulated inventory approaching a few molar — an upper bound set by the assumed Cu (Ⅱ) salt solubility ceiling. The ~10 s figure is therefore the time required to reach the assumed ceiling within the idealized model and is not a measured induction time. Beyond this point, additional Cu^2+^ is consumed by intermediate phase formation (consistent with the transient Raman signature reported in Section 2.5) and ultimately incorporated into the HKUST-1 framework as it crystallizes inward.

Taken together, these two stages define the concentration gradient around the NP. In the pre-shell stage, the gradient is a smooth diffusive profile decaying as 1/*r* from the surface (Eq. S3-1), whereas after shell formation the gradient becomes sharply localized across the shell, which acts as a diffusion barrier; the change from a shallow, extended gradient to a steep, shell-localized gradient is the proposed physical origin of the confinement-driven *S*_meta_ → *S*_labile_ transition. Under these idealized closed-cavity assumption, the corresponding time scale (on the order of ~10 s to approach the solubility ceiling) is consistent with the experimentally observed crystallization time of < 1 min (**Figure S24**) and the single-particle photooxidation time of ~60 s. Within this idealized picture, the system would operate near the practical concentration ceiling for most of the active crystallization period; the actual free-Cu^2+^ activity is expected to be substantially lower because of competing hydrolysis, precipitation, and coordination.

*Justification for the omission of the SBU-consumption term*

The mass balance in Eq. S3-4a includes a third term, -*k*_form_[Cu^2+^][BTC^3-^], representing the consumption of Cu^2+^ by SBU formation during HKUST-1 nucleation and growth. In the cavity concentration estimated in this Note, this term is deliberately omitted, so that the reported concentration represents the Cu^2+^ inventory available before an appreciable conversion to a solid framework. This deliberate simplification justified on three independent physical grounds; the resulting concentration should be interpreted as the instantaneous pre-nucleation driving force, not as a steady-state free-ion concentration sustained throughout crystallization.

(i) Timescale separation between Cu^2+^ accumulation and ligand supply. The same shell that confines Cu^2+^ within the cavity simultaneously restricts the inward transport of BTC^3-^ from the bulk. Because the shell is a strong diffusion barrier to ionic species, the rate-limiting reactant for SBU formation inside the cavity is the ligand, not the metal. During the burst phase immediately following shell closure, the cavity is therefore transiently Cu^2+^-rich and BTC^3-^-poor, while SBU formation has not yet consumed an a substantial fraction of the released metal. This transient window is proposed to provide the conditions that favor nucleation before substantial ligand-limited consumption occurs.

(ii) The driving force precedes consumption. The quantity relevant to classical nucleation theory is the chemical potential (supersaturation) at the moment of nucleation. SBU develops when Cu^2+^ ions overcome the nucleation barrier. Once nuclei form, they consume free Cu^2+^ and lower its concentration. Therefore, subtracting this post-nucleation conception from the pre-nucleation driving force would be physically inconsistent. The omission of the consumption term in the driving-force estimate is not merely a convenience but the correct treatment for the quantity of interest. The free-ion concentration after nucleation is necessarily low, and the difference corresponds precisely to the Cu^2+^ converted into solid HKUST-1.

(iii) Consumption and release are kinetically coupled. Following Le Chatelier’s principle, the consumption of free Cu^2+^ by SBU formation lowers the cavity concentration and thereby steepens the concentration gradient between the receding core surface and cavity, which in turn sustains continual Cu^2+^ release from the core. Therefore, generation and consumption are dynamically coupled rather than independent. The geometric and solid-state ceilings derived in this Note (43 M and 84 M, respectively) are determined purely by mass balance and are independent of the coupled kinetics; thus, they remain valid as absolute upper bounds regardless of the consumption rate.

A complete treatment incorporating the SBU-consumption term would require the determination of the rate constant *k*_form_, the reaction orders, and the intra-cavity concentration of BTC^3-^ (released from shell) under the high-ionic-strength, confined conditions of the cavity, none of which are independently constrained for this system. Introducing these as free parameters would increase the apparent precision of the model without improving its accuracy, and would obscure the limiting-case conclusion that shell confinement can raise the local Cu inventory by up to one to two orders of magnitude relative to conventional synthesis. We therefore adopted the consumption-free driving-force formulation and assumed that the free-ion concentration (buffered by both salt solubility and SBU consumption) lies below the reported values. This is a more conservative and transparent approach that focuses on the quantity robustly established by the analysis, i.e., the magnitude of S increase enabled by the shell formation.

**2.4. Comparison with conventional hydrothermal synthesis**

Because the absolute *K*_sp_ of HKUST-1 is poorly constrained in the aqueous conditions used here, we compare the present system to conventional hydrothermal synthesis using relative [Cu^2+^] ratios, which are independent of *K*_sp_.

**Table S4. Comparison of model-derived upper-bound local Cu inventories and nominal driving forces across reaction conditions. The post-shell values are limiting case estimates obtained under the assumptions of negligible leakage and consumption with an imposed Cu(II) salt solubility ceiling.** **They are not concentrations, free-Cu^2+^ activities, or thermodynamic supersaturations obtained from experimental measurements.**

| Condition | Local [Cu^2+^] | Temperature | Relative ratio* |
| --- | --- | --- | --- |
| Conventional hydrothermal | 50-100 mM | 110 °C | 1 (reference) |
| Pre-shell NP surface  (ensemble) | ~9 μM | 25 °C | ~10^-4^ |
| Pre-shell NP surface  (burst) | ~76 μM | 25 °C | ~10^-3^ |
| Post-shell cavity  (practical limit) | ~5 M  (saturation) | 25 °C | ~50–100 |
| Post-shell cavity  (geometric limit) | ~43 M  (supersaturation) | 25 °C | ~500 |

*** Relative ratio = [Cu^2+^]_condition_/[Cu^2+^]_hydrothermal_, with the hydrothermal [Cu^2+^] taken as a 75 mM (midpoint of the typical range)

Several key observations emerge from this comparison. First, the pre-shell stage alone offers no concentration advantage over conventional synthesis; in fact, the local [Cu^2+^] is three to four orders of magnitude lower. This explains why NPs without shells do not undergo rapid crystallization in our system. Second, within the limiting calculation the post-shell exhibits up to ~50–100 times the local Cu inventory of conventional hydrothermal synthesis at 110 °C at 25 °C. This upper-bound enrichment would arise from spatial confinement by the shell rather than from higher thermal energy. As an illustrative estimate of the temperature-equivalent driving force, for a fixed *K*_sp_, T·ln[Cu^2+^] compares the kinetic accessibility of nucleation across conditions. Using the ~5 M upper bound, this gives 338 K × ln0.075 ≈ -990 K for conventional synthesis versus 298 K × ln5 ≈ +480 K for the present system; the resulting difference (~1500 K) would correspond to a substantially larger nucleation driving force in the present system, providing a plausible kinetic rationale for rapid (< 1 min) ambient-temperature crystallization. This comparison is illustrative only, as $T\cdot\ln\left[ {Cu}^{2+} \right]$ is not a rigorous free-energy difference and neglects activity coefficients and the BTC^3−^ activity on which the true supersaturation also depends, and it inherits the assumed ~5 M ceiling. Third, the geometric upper bound of 43 M (corresponding to perfect confinement) provides a sanity check: even if Cu^2+^ does not encounter solubility limitations, the maximum concentration is physically bounded. The assumed practical limit (~5 M) is set by salt solubility rather than by geometric confinement, suggesting that chemistry rather than transport would bound the local inventory within the model.

**2.5. Self-consistency with experimental observations**

The closed-cavity analysis is consistent with multiple independent experimental observations:

(i) *Shell-dependent crystallization*. Rapid HKUST-1 formation occurs only for shell-bearing NPs (Section 2.6). This is consistent with the estimates: the pre-shell concentration is at most~76 μM, three orders of magnitude below that of a conventional synthesis system, whereas the post-shell cavity inventory exceeds that of conventional synthesis by up to 50–100 times under the closed-cavity assumption.

(ii) *Transient intermediate phases.* Raman spectroscopy and XRD results reveal an intermediate state distinct from both Cu_2_O and crystalline HKUST-1 (**Figure 3e**), with weakened Cu–O bonding and partial BTC coordination. Consistent with this, high-resolution TEM with FFT analysis shows that the shells contain both amorphous and crystalline domains, the latter indexing to Cu(Ⅱ) hydroxide (Cu(OH)_2_); the *d*-spacings of 0.371, 0.271, and 0.198 nm match the (021), (040), and (140)/(051)/(102) reflections, respectively, of orthorhombic Cu(OH)_2_ to within 3%, providing no evidence for crystalline Cu₂O in the analyzed regions. This coexistence of amorphous and partially crystalline hydroxide/carboxylate phases is the expected signature of the non-equilibrium *S*_meta_ regime, in which cavity Cu^2+^ concentrations approach the salt solubility limit and a distribution of transient intermediates forms before the final formation of HKUST-1.

(iii) *Crystallinity ladder from S_meta_ to S_labile_.* The trend in local enrichment implied by this analysis agrees with the degree of crystallinity of the product, providing a qualitative support for the proposed local concentration field rather than an independent measurement of it. At the core–shell stage with modest *S*_meta_ enrichment, powder XRD patterns (**Figure 3d**, Path A) exhibit the full set of HKUST-1 reflections at their expected positions, including (111), (200), (220), (222), (400), (511), (440), and (600), but with low intensity and pronounced broadening relative to the pristine product. This identifies the core–shell intermediate as poorly crystalline HKUST-1 rather than a fully developed framework, in agreement with the heterogenous amorphous/crystalline structure revealed by high-resolution TEM and the multi-component Raman signature. As continual self-photooxidation drives the cavity from the *S*_meta_ to *S*_labile_ regime, this poorly crystalline material develops into the highly crystalline octahedral nano-HKUST-1 final product, which exhibited a diffraction pattern with sharp, intense reflections (**Figure 4e**). Therefore, the progression from a poorly crystalline framework to a highly crystalline single phase is consistent with the proposed *S*_meta_ → *S*_labile_ transition, although it does not independently quantify the local supersaturation or define the boundary between the two regimes quantitatively.

(iv) *Loss of the HKUST-1 pathway under delocalization.* The analysis also implies that disrupting the localized concentration field should suppress HKUST-1 formation. When supersaturation is delocalized by stirring (Path B in **Figure 3b and c**), the product is not HKUST-1 but a distinct crystalline phase. The powder XRD pattern shows a single strong reflection at 2θ ≈ 9.96° (*d* ≈ 8.87 Å) that does not correspond to any reflection of HKUST-1, indicating that the HKUST-1 framework did not form. This phase, together with the bar- and whisker-like morphology observed by TEM, is consistent with a one-dimensional Cu-BTC coordination chain of Cu(BTC)·3H_2_O (cf. *Mater. Adv.* 2022, 3, 224), which forms when ligand deprotonation and local supersaturation are insufficient to access the three-dimensional HKUST-1 framework. This off-pathway outcome may suggest that localization strongly biases the system toward HKUST-1 crystallization under the tested conditions, although the single transport perturbation does not by itself establish this conclusion.

(v) *Crystallization time scale.* Within the idealized closed-cavity model, the accumulation rate implies that the assumed solubility ceiling would be achieved within ~10 s after shell formation, with crystallization substantially complete within ~60 s. This model time scale is compatible with the ensemble time scale observed experimentally (**Figure S24**); however, the trajectory of an individual particle needs further investigation.

(vi) *Final particle size*. The size distribution of the nano-HKUST-1 crystals closely matches that of the parent t‑Cu_2_O (**Figure S21**), indicating that crystal growth proceeds predominantly inward from the shell rather than outward into the bulk. This is consistent with the closed-cavity model, although contributions from local dissolution and reprecipitation in the shell-bounded region cannot be excluded.

**2.6. Assumptions and limitations**

The above analysis relies on the following assumptions:

(i) *Strongly restricted transport across the shell*. This is supported, but not established, by two experimental observations (the ICP-OES dissolved-Cu measurement and the absence of detectable post-shell metal incorporation; Section 2.2). Neither observation constitutes a complete Cu mass balance or a quantitative permeability measurement, and the shell is not claimed to be impermeable. Any residual permeability would reduce the cavity inventory below the assumed solubility ceiling but would not change the qualitative expectation that the shell increases the local Cu^2+^ inventory relative to pre-shell levels.

(ii) *Spherical symmetry of the NP, shell, and cavity.* Faceted NPs (e.g., t-Cu_2_O) exhibit higher *J* values at hole-accumulating facets ((100), **Figure S5**), locally increasing the concentration beyond the spherical estimate, which is considered a conservative lower bound.

(iii) *Treatment of Cu^2+^ salt solubility as a hard ceiling.* The actual solubility under the tested conditions depends on counterion identity, pH, ionic strength, and the presence of organic ligands (BTC, TEA). The 5 M value used here is an order-of-magnitude estimate based on common Cu(Ⅱ) salts, whereas the actual ceiling may differ by a factor 2–3 in either direction without changing the qualitative conclusions.

We further note that CuCl_2_ and Cu(NO_3_)_2_ are not the species present in the Cu_2_O/BTC/TEA system; their solubilities serve only as an order-of-magnitude proxy for the Cu(Ⅱ) saturation regime. The true free-Cu^2+^ activity is instead governed by hydrolysis, complexation, Cu(OH)_2_ precipitation, and Cu-carboxylate formation, and the relevant supersaturation depends on the activities of both the Cu and BTC species rather than on [Cu^2+^] alone. Accordingly, the concentration quoted throughout this Note are hypothetical upper limits rather than measured values.

(iv) *Activity coefficients at high ionic strength.* At cavity concentrations of 1–5 M, the Debye–Hückel approximation fails, and activity coefficients deviate substantially from unity. Thus, the effective driving force for nucleation may differ from the nominal concentration ratio by a factor of order unity, absorbed by the order-of-magnitude framing used throughout.

(v) *Choice of relative ratios over absolute S values.* The absolute supersaturation *S* = [Cu^2+^]/[Cu^2+^]_sat_, in which [Cu^2+^]_sat_ depends on the *K*_sp_ of HKUST-1 under the specific test conditions. The relative-ratio framing used here is independent of *K*_sp_ and is therefore more robust, while still capturing the essential physics of the shell-mediated *S*.

Despite these simplifications, the analysis provides a limiting-case, order-of-magnitude framework that is consistent with the central hypothesis of the present study: the shell-restricted transport allows photogenerated Cu to accumulate locally and thereby support ambient-temperature crystallization of HKUST-1 in water. The upper-bound enrichment (up to ~50–100 times) relative to conventional hydrothermal synthesis (**Table S4**), together with the transport observation of Section 2.2, offers a plausible rationale for crystallization within ~1 min at 25 °C, whereas conventional approaches requires hours at higher temperature in organic solvents. However, our analysis does not provide experimental results for the local Cu^2+^ concentration, the free-Cu^2+^ activity, or the thermodynamic supersaturation of the present system, nor does it quantitatively define the *S*_meta_-to-*S*_labile_ transition. These aspects will be investigated in future work.

**Supporting Note 3. Mechanistic interpretation of solvent-mediated interfacial precipitation of HKUST-1: Protonation-induced solubility suppression and morphological regulation**

HKUST-1 is a copper-trimesic acid (Cu-BTC) coordination polymer with poor intrinsic colloidal dispersibility in water. However, during aqueous-phase synthesis in the presence of TEA, the BTC ligand remains partially deprotonated and stabilized by residual TEA, forming a hydration layer around the growing crystals. This electrostatic and hydration-based stabilization prevents aggregation, yielding a metastable colloidal suspension. Notably, these particles cannot be sedimented even under ultracentrifugation (10,000 rpm), demonstrating strong colloidal stability and precluding mechanical separation. However, over time, hydrolytic degradation of the framework occurs due to BTC dissociation (*k*_dissociation_>0), especially under ligand-excess and basic conditions. Therefore, a purification strategy is expected to chemically quench framework evolution and induce precipitation for product recovery.

We implemented a solvent-mediated interfacial purification method by layering alcohols of various polarities and hydrogen-bonding capabilities on top of the aqueous reaction phase. This triggers the following sequence.

1. **Partition-driven extraction of basic and organic components**

TEA (log*P* ≈ +0.2) and BTC (log*P* ≈ -0.9) undergo spontaneous phase separation across the aqueous–alcohol interface, where *P* = [solute]_octanol_/[solute]_water_. More hydrophobic TEA preferentially partitions into the alcohol phase. This is supported by UV-Vis spectra showing a characteristic peak at ~280 nm in the alcohol layer, corresponding to TEA. Notably, the A_280 nm_ peak intensity after TEA extraction follows the order of methanol > ethanol > propanol. This order reflects the solvent polarity and TEA solubility. Partitioning of BTC also occurs to a lesser extent, especially in polar solvent, further altering local chemical equilibrium.

2. **Local pH gradient and ligand protonation**

Following TEA extraction, the bulk aqueous pH changes only slightly (5.44 to 6.00) while the alcohol layer becomes mildly alkaline (8.2 for ethanol). In addition to the small pH shift during TEA extraction, the basic species that maintain the surface carboxylates of HKUST- 1 in their deprotonated, colloidally stabilized state were removed. As TEA partitions into the alcohol phase, the accompanying interfacial ion redistribution locally modulates the proton activity at the aqueous-alcohol interface, shifting the effective equilibrium of the surface-bound carboxylate groups toward protonation:

$$R-\mathrm{COO}^{-}+H^{+}\leftrightarrow R-COOH \left( \mathrm{effective} K_{a3}\approx{10}^{-6} \mathrm{for} the surface bound carboxylate \right)$$

Here, the equilibrium constant refers to the carboxylate groups coordinated at the crystal surface, whose effective acidity differs from that of free trimesic acid owing to metal coordination and the local interfacial environment. The protonation of these surface carboxylates reduces their negative charge and weakens the hydration-based stabilization, decreasing the colloidal stability of the particles. The chemical potential of the colloid decreases upon neutralization: $\text{∆}\text{G}_{\text{solubility}}\text{∝}\text{μ}_{\text{solvated}}\text{-}\text{μ}_{\text{protonated}}\text{<0}$. Consequently, HKUST-1 particles aggregate and precipitate near the interface.

3. **Solvent-specific morphological outcomes**

Due to the unique dielectric constant (ε), hydrogen-bond donating/accepting ability, and viscosity (η) of each solvent, various crystal morphologies are obtained, which in turn affect diffusion and surface protonation kinetics. The observed trends are listed in the table below.

**Table S5. Solvent-dependent extraction efficiency and resulting crystal morphology**

| **Solvent** | **TEA extraction** | **Crystal morphology** | **Interpretation** |
| --- | --- | --- | --- |
| Methanol | High |  | High TEA extraction  → rapid charge neutralization  → uncontrolled aggregation  High polarity and low viscosity accelerate diffusion but impair size control. |
| Ethanol | Moderate |  | Balanced TEA extraction and moderate H-bonding afford optimal quenching and controlled precipitation. Ethanol provides sufficient polarity and moderate proton mobility for quenching. |
| Propanol | Low |  | Slower TEA extraction and lower proton transfer efficiency lead to partial charge neutralization. Incomplete precipitation promotes the formation of defects, dissociation, and possible Cu^2+^ clustering. |
| Acetone | Not  detectable |  | No hydrogen bonding and poor miscibility prevent proton transfer, resulting in etching and dissociation on the surface. |
| Hexane | None | None | Immiscible and non-polar; no interfacial transport occurs. TEA/BTC remain in aqueous phase; no quenching or precipitation. |

This interfacial quenching strategy achieves three objectives:

1. Terminates crystallization/dissociation by removing basic and ligand species from the aqueous phase.

2. Triggers protonation-driven destabilization of colloidal HKUST-1, enabling purification without centrifugation.

3. Allows morphological tuning via solvent-mediated control of interfacial chemistry.

This mechanistic framework demonstrates the collaborative governing effects of solvent polarity, protic character, and diffusion rates on interfacial quenching and purification. Our findings highlight the interplay between p*K*a-controlled protonation and solvent engineering as a rational tool for crystal purification and design under mild conditions.

**Supporting Note 4. Design criteria for linker generalization**

This note examines a candidate physical descriptor to determine whether a given linker is compatible with the localized supersaturation strategy. Among the three linkers examined, the observed outcomes correlate with a single requirement: the fully deprotonated anionic linker must approach the negatively charged Cu_2_O surface to nucleate the confining shell. Where the shell formed, crystalline domains of the corresponding framework were subsequently identified, for HKUST-1 (from H_3_BTC) and Cu–BDC (from H_2_BDC). On this basis, the strategy may extend to other linkers that retain sufficient near-surface accessibility, although this expectation has not been tested beyond the linkers reported here. The descriptor is developed below by deriving a characteristic energy scale from the Boltzmann distribution and comparing it with the outcomes across the linkers and Cu_2_O facets examined in this study; the comparison is a correlation over a small data set rather than a validated first-principles design rule. Note that this accessibility criterion concerns linker access before shell formation and is distinct from the *C*_critical_ presented in Supporting Note 2, which governs Cu^2+^ nucleation inside the already-formed cavity; the two criteria are relevant for different chemical species at different stages and are treated independently.

.

**4.1. Electrostatic accessibility criterion**

Shell formation requires a fully deprotonated, anionic linker to approach and accumulate at the negatively charged Cu_2_O surface. We make this requirement quantitative in three steps.

**(1) Surface concentration from the Boltzmann distribution**

In the diffuse part of the electrical double layer, the local concentration of an ion with valence *z* at a surface held at electrostatic potential *ψ*_0_ follows the Boltzmann distribution relative to the bulk:

| $\frac{C_{\mathrm{surf}}}{C_{\mathrm{bulk}}}=\exp\left( -\frac{ze\psi_{0}}{kT} \right)=e^{-W}, \mathrm{where} W\equiv\frac{ze\psi_{0}}{kT}$ | (S4-1) |
| --- | --- |

Here, *e* is the elementary charge (1.602 × 10^-19^ C), *k* is Boltzmann’s constant, temperature *T* is 298 K, and *W* is the electrostatic exclusion energy in units of *kT*. For an anion (*z* < 0) at a negatively charged surface (*ψ*_0_ < 0), *W* >0 and *C*_surf_/*C*_bulk_ < 1: the near-surface electrostatic depletion of the linker increases as its charge becomes more negative. The expression in Eq. S4-1 is the standard mean-field result for an ion in the diffuse layer and contains no adjustable parameter; it is a smooth, monotonic function of *W,* and accessibility declines continuously as *W* increases, with no intrinsic discontinuity.

**(2) Thermal energy sets the characteristic scale of the transition.**

Although accessibility varies continuously, it changes most steeply when *W* is comparable to the thermal energy. When *W* ≪ 1, the barrier is negligible, and the surface concentration approaches the bulk value; when *W* ≫ 1 the surface is strongly depleted. The crossover between these regimes occurs at $W\approx1$:

| $W\approx1\Leftrightarrow\frac{C_{surf}}{C_{bulk}}\approx\frac{1}{e}=0.37$ | (S4-2) |
| --- | --- |

This value is not an empirical cut-off fitted to the data, nor a sharp boundary. It is a point at which the *W* equals the thermal energy, the only intrinsic energy scale in the problem, suggesting that the surface concentration falls 1/*e* of the bulk, an exact consequence of the Boltzmann expression in Step 1. Therefore, we use *W ≈* 1 (equivalently *C*_surf_/*C*_bulk_ *≈* 1/*e*) only as a characteristic reference scale, not as a predictive accessible/excluded boundary; accessibility declines continuously through this region rather than switching abruptly.

**(3) Evaluation of the three linkers**

We evaluated the nominal accessibility at the t-Cu_2_O surface used throughout the study, using the measured zeta potential (ζ = -7.05 mV; **Figure 2g**) only as an electrokinetic input in place of the surface potential $\psi_{0}$. Because ζ is defined at the slipping plane not on the surface, it is not equivalent to $\psi_{0}$; the resulting values are model-derived ratios rather than measured near-surface concentration, and they scale with the assumed interfacial potential. With *kT*$=$ 25.7 meV at 298 K, substituting the fully deprotonated charged BDC^2−^, BTC^3−^, and DHTP^4−^ into *W* = *zeψ*_0_/*kT* gives:

BDC ($z=-2$): $\frac{ze\psi_{0}}{kT}=\frac{\left( -2 \right)\left( -7.05 mV \right)}{25.7 mV}=+0.549$; $\frac{C_{\mathrm{surf}}}{C_{\mathrm{bulk}}}=\exp\left( -0.549 \right)=0.578$

BTC ($z=-3$): $\frac{ze\psi_{0}}{kT}=\frac{\left( -3 \right)\left( -7.05 mV \right)}{25.7 mV}=+0.823$; $\frac{C_{\mathrm{surf}}}{C_{\mathrm{bulk}}}=\exp\left( -0.823 \right)=0.439$

DHTP ($z=-4$): $\frac{ze\psi_{0}}{kT}=\frac{\left( -4 \right)\left( -7.05 mV \right)}{25.7 mV}=+1.098$; $\frac{C_{\mathrm{surf}}}{C_{\mathrm{bulk}}}=\exp\left( -1.098 \right)=0.334$

The near-surface linker concentration falls to 58%, 44%, and 33% of the bulk value for BDC, BTC, and DHTP, corresponding to surface depletions of 42%, 56%, and 67%, respectively. Each additional unit of linker charge multiplies the exclusion factor by exp(*eψ*_0_/*kT*) = exp(-0.21) = 0.76, i.e. a further ~25% reduction in surface concentration per unit charge at this surface potential.

Compared with the characteristic scale of Step 2, the value of BTC sits just inside the accessible regime *(W* = 0.82 < 1; *C*_surf_/*C*_bulk_ = 0.44 > 1/*e*), which corresponds to shell formation, whereas DHTP sits just beyond the crossover (*W* = 1.10 > 1; *C*_surf_/*C*_bulk_ = 0.33 < 1/*e*) with no shell formation. The change in behavior between BTC and DHTP therefore brackets the *W ≈* 1 scale. However, this observation is based on only three linkers and cannot be distinguished from an interpolation between two measured points, and it should not be read as validation of the scale. In line with this continuous description, BDC (the most accessible of the three linkers; *W =* 0.55) readily forms a shell, whereas DHTP lies only modestly beyond the crossover rather than being excluded by a large margin. Because ζ is used to represent *ψ*_0_, the absolute accessibility values scale with the assumed surface potential. The ordering BDC > BTC > DHTP is invariant under such a rescaling, whereas the position of the crossover relative to the individual liners is not. The observed shell-formation outcomes are consistent with the *W ≈* 1 scale under the ζ *≈* *ψ*_0_ approximation adopted here, however, this agreement does not independently establish the validity of the scale.

**4.2. Independent validation across facets**

Because *W* depends on *ψ*_0_, the same scale defines a nominal surface potential for each linker at which accessibility passes the reference:

| $\left\vert\psi_{0} \right\vert\approx\frac{kT}{\left\vert z \right\vert e}$ | (S4-3) |
| --- | --- |

This gives approximate values of 12.8 mV for BDC, 8.6 mV for BTC, and 6.4 mV for DHTP. This trend is supported by the facet experiments. BTC forms a proper shell on t-Cu_2_O (|$\psi_{0}$| = 7.05 mV, below its crossover of 8.6 mV) but does not form a shell on the more strongly charged c-Cu_2_O (26.1 mV, far above the crossover), where *W* = 3.0 *kT* drives *C*_surf_/*C*_bulk_ down to 0.05. DHTP has a higher approximate value than its crossover on t-Cu_2_O (7.05 mV > 6.4 mV) and forms no shell, whereas its accessibility rises to 0.72 on weakly charged o-Cu_2_O (2.09 mV, below 6.4 m) with shell formation. Therefore, a single characteristic scale *W ≈ 1* is consistent with the shell-formation outcomes across the linker-facet combinations examined here, indicating that near-surface accessibility is an important variable. Linker-specific coordination and growth properties were not independently varied and therefore cannot be excluded as contributing factors. These crossover potentials are stated as testable predictions: changing $\psi_{0}$, facet, pH, or ionic strength should move a given linker across the reference.

**4.3. Scope and limitations**

For accessibility study, each linker occupies a position along the accessibility axis, and the characteristic scale (*C*_surf_/*C*_bulk_ ≈ 1/*e*) separates the accessible region, where the shell forms followed by inward crystallization from the excluded region. BDC and BTC lie in the accessible region and produce Cu–BDC-like and HKUST-1, respectively, whereas DHTP lies in the excluded region. This provides a working correlation rather than a design rule: a Cu-MOF linker may be a promising candidate when its nominal electrostatic accessibility lies on the favorable side of the 1/*e* scale at an operative surface potential (equivalently, *W = zeψ*_0_/*kT* below ~ 1). This quantity can be evaluated for different linker–facet pairs based on the charge of the linker and the measured *ψ*_0_, and the surface potential itself can be tuned via the facet type, pH, and ionic strength, providing a route to moving otherwise-excluded linkers (such as DHTP) into the accessible region, as demonstrated by the thin shell formed by DHTP on Cu_2_O.

We note three limitations for this accessibility study. First, the descriptor is derived from a mean-field Boltzmann description that treats the linker as a point charge, without considering finite ion size, double-layer screening at the relevant ionic strength, linker speciation and partial deprotonation specific chemical adsorption, and direct surface coordination. The *W ≈ 1* crossover is therefore a characteristic scale accurate to the order of *kT*, and the agreement across the linkers and facets examined in this study may not be generalized. Second, *ζ* is used as a surrogate unput for *ψ*_0_; because *ζ* is defined at the slipping plane, the absolute accessibility values and the position of any individual linker relative to the 1/*e* reference depend on this substitution, although the ordering of the linkers at a fixed negative potential does not. Third, the descriptor has been evaluated for only three linkers and three facets. A wider library should be considered before the 1/*e* reference is used for prediction. Within these bounds, the analysis provides a physically motivated correlation that is consistent with the observed outcomes and that may help identify candidate linkers and surface conditions for extending the strategy. It is not a validated predictive design rule, and the BDC-derived product was characterized only at the level of locally identified crystalline domains, without determination of bulk phase, purity, yield, or porosity.

**Supporting Experimental Section**

**Optical microscopy**

Dark-field microscopy was performed using an inverted microscope (Nikon, ECLPSE Ti2-E) equipped with 100× oil-immersion objective lens (Nikon, 1.49 NA, CFI SR HP Apochromat TIRF). The microscope setup included a motorized stage (TI2-S-SE-E), a perfect focus system (TI2-N-ND-P), and an electron multiplying charge-coupled device (Andor, iXorn Ultra 897). Illumination was provided by a mercury lamp (Nikon, C-HGFIE Intensilight), and the light source power was calibrated using a power meter connected to a photodiode sensor (Thorlabs, PM100D, S120VC).

**Observation of photodecomposition**

The photodecomposition of Cu_2_O NPs was monitored by analyzing changes in scattering intensity using dark-field microscopy. A 35 mm MatTek dish was cleaned with Alconox and NaOH before loading the Cu_2_O NPs dispersed in DI water onto the dish. The reactions were recorded as video files using NIS-Elements software (Nikon) and subsequently analyzed with ImageJ software.

**Characterization instruments**

The synthesized NPs and HKUST-1 products were characterized using FE-SEM (Hitachi, S-4800) operated at an acceleration voltage of 3.0 kV and field-emission TEM (Hitachi, HF-3300) at 300 kV. An XRD system (Panalytical, Empyrean) equipped with Cu Kα radiation and an FT-IR spectrometer (Thermo Scientific, Nicolet Continuum) were used to analyze structural and functional groups. The electronic structures were investigated using XPS (Thermo Scientific, ESCALAB 250Xi), and UV-Vis spectroscopy (Agilent, Cary 8454). Optical bandgaps were calculated using Tauc plots. The concentration of Cu^2+^ ions was quantitatively determined using an inductively coupled plasma-mass spectrometer (ICP-MS, PerkinElmer, NexION 2000) and ICP-OES (PerkinElmer, Acio500) . The surface charge of the NPs was measured using a Zetasizer Nano series (Malvern, MAL1160456), and the Brunauer–Emmett–Teller surface area was analyzed by nitrogen adsorption experiments (details). Raman spectroscopy. Temperature change were monitored with a thermal imaging camera (FLIR E8 Pro).

**Sample preparation for characterization**

Cu_2_O NPs and HKUST-1 were loaded onto 3×3 mm Si wafers for SEM, UPS, and XPS analyses. For SEM imaging, the samples were sputter-coated with platinum using a current of 15 mA for 15 sec. HKUST-1 samples for TEM analysis were deposited onto Ni grids, while samples for XRD analysis were loaded onto 5×5 mm glass substrates, prepared with ethanol as the solvent to ensure thick and uniform deposition. For FT-IR, Raman spectroscopy, and BET analysis, HKUST-1 was prepared in solid state by vacuum drying. For UV-Vis spectroscopy, Cu_2_O NPs solution was measured in a quartz cuvette with DI water as the blank reference to determine its absorbance. For ICP-MS and ICP-OES analysis, the Cu_2_O NPs was centrifuged, the supernatant was acidified with aqua regia (HCl:HNO_3_ = 3:1), followed by dilution with DI water. The surface charge of the Cu_2_O NPs was measured using a Zetasizer Nano series; Cu_2_O NPs were dispersed in DI water and measured at 25 °C with a Malvern DTS1070 cuvette.

**Self-fabricated reaction holder**

To ensure consistent experimental conditions, a custom holder was fabricated to securely position the samples. The holder was designed to maintain a fixed distance between the light source and sample. The design process was conducted using Fusion 360 (Autodesk) software, and the holder was fabricated using an FDM-type 3D printer (Ultimaker 2+ Connect) with a commercially available polylactic acid filament (Ultimaker) (**Figure S8**).

**References**

[1]	J. Kang, Y. Lee, S. Lee, et al., “Dynamic Three-Dimensional Structures of a Metal–Organic Framework Captured with Femtosecond Serial Crystallography,” *Nature Chemistry* **16** (2024): 693–699.
https://doi.org/10.1038/s41557-024-01460-w

[2]	T. Prohaska, J. Irrgeher, J. Benefield, et al., “Standard Atomic Weights of the Elements 2021 (IUPAC Technical Report),” *Pure and Applied Chemistry* **94**, no. 5 (2022): 573–600.
https://doi.org/10.1515/pac-2019-0603

[3]	A. Kirfel and K. Eichhorn, “Accurate Structure Analysis with Synchrotron Radiation. The Electron Density in Al₂O₃ and Cu₂O,” *Acta Crystallographica Section A* **46**, no. 4 (1990): 271–284.
https://doi.org/10.1107/S0108767389012596

[4]	P. Vanýsek, “Ionic Conductivity and Diffusion at Infinite Dilution,” in *CRC Handbook of Chemistry and Physics*, 92nd ed., edited by W. M. Haynes, Boca Raton, FL: CRC Press, 2011, pp. 5-77–5-79. ISBN 978-1-4398-5511-9.

[5]	R. A. Robinson and R. H. Stokes, *Electrolyte Solutions*, 2nd rev. ed., Mineola, NY: Dover Publications, 2002, 608 pp. ISBN 978-0-486-42225-1.

[6]	D. Kashchiev, *Nucleation: Basic Theory with Applications*, Oxford: Butterworth-Heinemann, 2000. ISBN 978-0-7506-4682-6.

[7]	J. W. Mullin, *Crystallization*, 4th ed., Oxford: Butterworth-Heinemann, 2001. ISBN 978-0-7506-4833-2.

[8]	Y. Ahn, M. Park, J. Jang et al., “Spatiotemporal Antibacterial Strategy via Intra-Particle Charge Transfer-Enhanced Self-Photooxidation of Cu2O Nanoparticles,” Chemical Engineering Journal **520** (2025): 165461.

https://doi.org/10.1016/j.cej.2025.165461

[9]	R. C. Weast, ed., *CRC Handbook of Chemistry and Physics*, 68th ed., CRC Press, Boca Raton, FL, USA, 1987–1988, p. 88-89.

[10]	V. K. Filippov, D. S. Barkov, and Yu. A. Fedorov, “Applcation of the Pitzer Equations to the Solubility of Ternary Aqueous Nitrate Solutions at 25 °C,” Journal of Solution Chemistry **15**, no. 7 (1986): 611–619.

https://doi.org/10.1007/BF00645814

[11]	D. Sibarani, H. Sippola, P. Taskinen, et al., “Critical Evaluation of CuSO_4_–H_2_O System up to Solubility Limit, from Eutectic Point to 373.15 K,” Chemical Engineering Science **257**, no. 10 (2022): 117689.

https://doi.org/10.1016/j.ces.2022.117689
